# An adhesive locomotion model for the rock-climbing fish, *Beaufortia kweichowensis*

**DOI:** 10.1038/s41598-019-53027-2

**Published:** 2019-11-12

**Authors:** Jinrong Wang, Chen Ji, Wei Wang, Jun Zou, Huayong Yang, Min Pan

**Affiliations:** 1 State Key Laboratory of Fluid Power and Mechatronic Systems, Zhejiang University, Hangzhou, 310027 China; 2 Institute of Marine Science and Technology, Shandong University, Qingdao, 266237 China; 3 Center for Power Transmission and Motion Control, Department of Mechanical Engineering, University of Bath, Bath, BA2 7AY UK

**Keywords:** Animal behaviour, Animal behaviour, Biomechanics, Biomechanics

## Abstract

The rock-climbing fish (*Beaufortia kweichowensis*) adheres to slippery, fouled surfaces and crawls both forward and backward in torrential streams. During locomotion, two suckers can be distinguished. Here, the general skeletal structure of the rock-climbing fish was determined using microtomography. Friction and adhesion were positively correlated, as were friction and fin ray angle. The unique adhesive locomotion system used by the rock-climbing fish was observed with a high speed camera. This system comprised two anisotropic suckers bearing two paired fins and two girdle muscles. A locomotion model was established based on these results. In this model, the fin states controlled the direction of motion using anisotropic friction, and alternate contractions of the girdle muscles provided propulsion during bidirectional crawling. This adhesive locomotion system was compared with other biological locomotion mechanisms. Based on these comparisons, we hypothesized that this novel system might represent an energy-saving solution for undulatory underwater vertical movement without detaching from the substrate.

## Introduction

Small fishes that live in fast-flowing water face an extreme environment, with strong currents and little food^1,2^. Some of these fishes have evolved abilities such as station-holding (maintaining position) and locomotion to prevent dislodgement^1^. Station-holding fish such as gobies^3–6^, catfishes^7,8^, clingfishes^2,9^, and remoras^10–12^ have also evolved adhesive apparatuses which act like suckers, which use negative pressure to attach to the substrate. Various fish locomotion mechanisms have been reported in the literature^3^. For example, some gobies (*Sicyopterus stimpsoni*)^4^ and catfishes (*Astroblepus* sp. “Peru”)^7^ use oral and pelvic suckers to climb surfaces without axial undulation (“inching up“^4^), while some gobies (juvenile *Awaous guamensis*)^4^ climb using powerful bursts of eel-like axial undulations^3,4,13^. Some fishes are able to walk underwater or on land using their pectoral fins and pelvic fins such as frogfish^14^, mudskippers^15^, lungfish^16^ and walking catfish^17^ by using their tail to push their bodies forward, pivoting over the pectoral fins. The cavefish (*Cryptotora thamicola*) use a diagonal-couplet lateral sequence gait, which is accomplished by rotation of the pectoral and pelvic girdles^18^. Notably, none of these fishes walk with a continuous contact with the substrate because they need to lift up their fins. *Beaufortia kweichowensis* (Fang, 1931), locally known as “the rock-climbing fish“^19^, has a powerful adhesive system with anisotropic properties^20^. When *B. kweichowensis* remain stationary, the whole body acts as a sucker. However, during locomotion, two suckers of *B. kweichowensis* can be distinguished: an anterior sucker bearing the pectoral fins and the head, and a posterior sucker bearing the pelvic fins. Although these sucker components are similar to those of Balitoridae like *Beaufortia leveretti*, which is a different species from the same genus^1^, the locomotion mechanisms of the rock-climbing fish remain unclear.

In this study, we investigated the function of the mobile elements of *B. kweichowensis* fin ray by examining general skeletal structure using microtomography (µCT), and by determining the influences of friction on fins using freshly euthanized fish. To better understand locomotive mechanisms, we also used a high-speed camera to analyze the kinematics of live fish. Although unculi^21–23^ on paired-fin pads were reported in loaches (Balitorinae), few locomotive studies have been performed since 1970^24^. Previous studies have suggested that a left-right undulation of the trunk using two alternate suckers constituted a locomotion mechanism^1^, but no functional evidence for this mechanism has been presented. Based on our experimental results, we described herein a unique locomotion system, where an adhesive-undulatory mechanism is used for crawling. However, because no electromyographic or pressure distribution testing was performed, the functions of the girdle and fin muscles during these modes of locomotion remain hypothetical. Nonetheless, our kinematic analysis allowed us to propose functional theories regarding the locomotive mechanisms of *B. kweichowensis*. This locomotion system consisted of two anisotropic suckers bearing two paired fins and two girdle muscles. During bidirectional crawling, the fins controlled the direction of motion and the girdle muscles provided power. This adhesive locomotion system was effective on underwater vertical surfaces, and allowed the fish to move while remaining adhered to the substrate. Thus, this mechanism may represent an energy-saving biomimetic solution to the problem of climbing without detaching from vertical surfaces.

## Results

### Ventral girdle and fin

**Figure 1:**
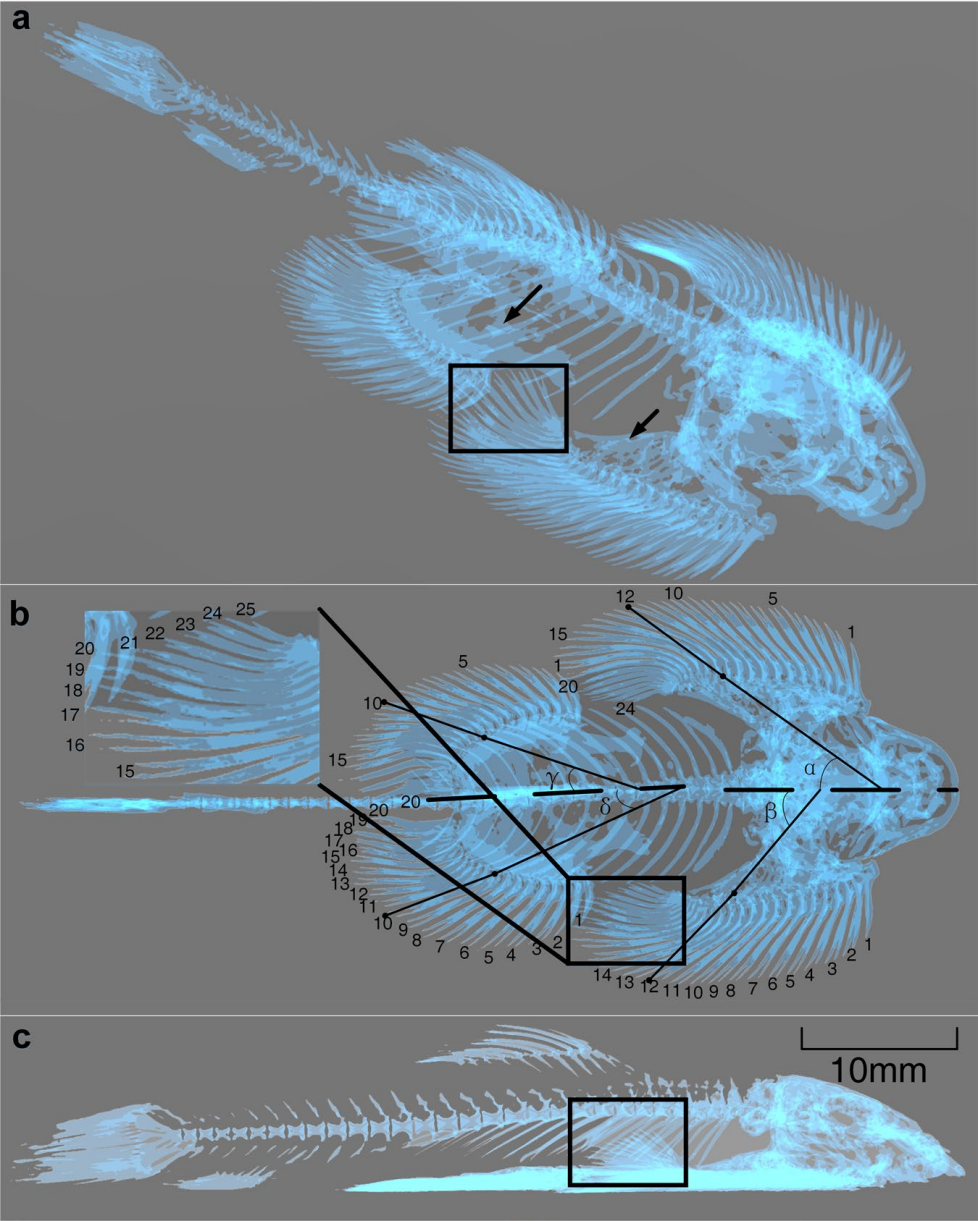
3D microtomographic (µCT) reconstruction of *Beaufortia kweichowensis*. (**a**) Axonometric view of the 3D model. The two black arrows point to the bony plates; the pectoral fins overlap the pelvic fins within the box. (**b**) Dorsal view of the 3D model. (**c**) Lateral view of the 3D model. The posterior pectoral fin rays overlap the anterior pelvic fins within the box. All images were taken of euthanized specimens, without further treatment. The pictures were processed by 3D Viewer in Windows 10.

Using µCT, we identified two plates on the ventral surface of *B. kweichowensis*: the pectoral and pelvic plate (Fig. 1a). These plates were not connected. The bones of the pectoral girdle formed a bony pectoral plate. There were 24 to 26 pectoral fin rays on either side of the body, with the first fin ray being unbranched (n = 10), and 24 or 25 pectoral fin rays in Fig. 1b. The anterior fin rays, usually in contact with the substrate, were thickened. The posterior fin rays, adducted to the body and covering the anterior pelvic fin, were thin. The pelvic girdle consisted of two ossified basipterygia, which formed a bony pelvic plate. There were 19 to 20 pelvic fin rays on either side of the body, with the first fin ray being unbranched (n = 10), and 20 pelvic fin rays in Fig. 1b. All fin rays were usually in contact with the substrate. The pectoral fins overlapped the pelvic fins (Fig. 1c).

### Friction coefficient

**Figure 2:**
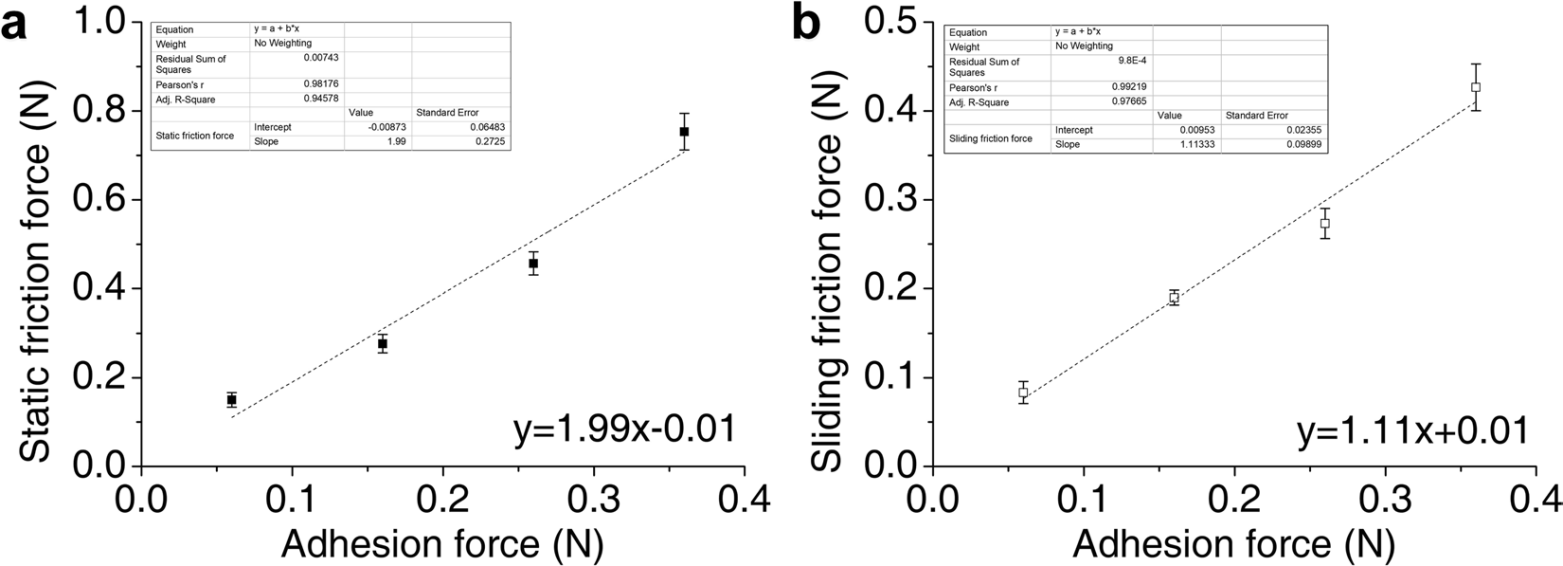
Relationships between friction forces and adhesion forces on an underwater substrate. (**a**) Static friction force at various adhesion forces. The expected relationship between static friction and adhesion force is indicated by a dash line. The slope of the dashed line is equivalent to the static friction coefficient. (**b**) Sliding friction force at different adhesion forces. The expected relationship between sliding friction and adhesion force is indicated by a dashed line. The slope of the dashed line is equivalent to the sliding friction coefficient. (n = 3, Mean ± SD).

In a rushing stream, friction in the opposite direction of the dragging force helps the fish to adhere. However, friction will also oppose forward locomotion. Here, we tested friction force against various adhesion forces. In every pulling test, we considered the maximum pulling force before movement in the initial phase as static friction, and the subsequent stable pulling force as sliding friction. We performed three pulling tests for each adhesion force. As the adhesion force increased from 0.06 to 0.36 g, static friction force and sliding friction force increased almost proportionally (Fig. 2) (Supplementary Figs 2 and 3). We found that the coefficients of static friction (1.99 ± 0.27) and sliding friction (1.11 ± 0.10) by linear fitting in OriginPro 9 remained nearly constant.

### Friction force and fin ray angle

**Figure 3:**
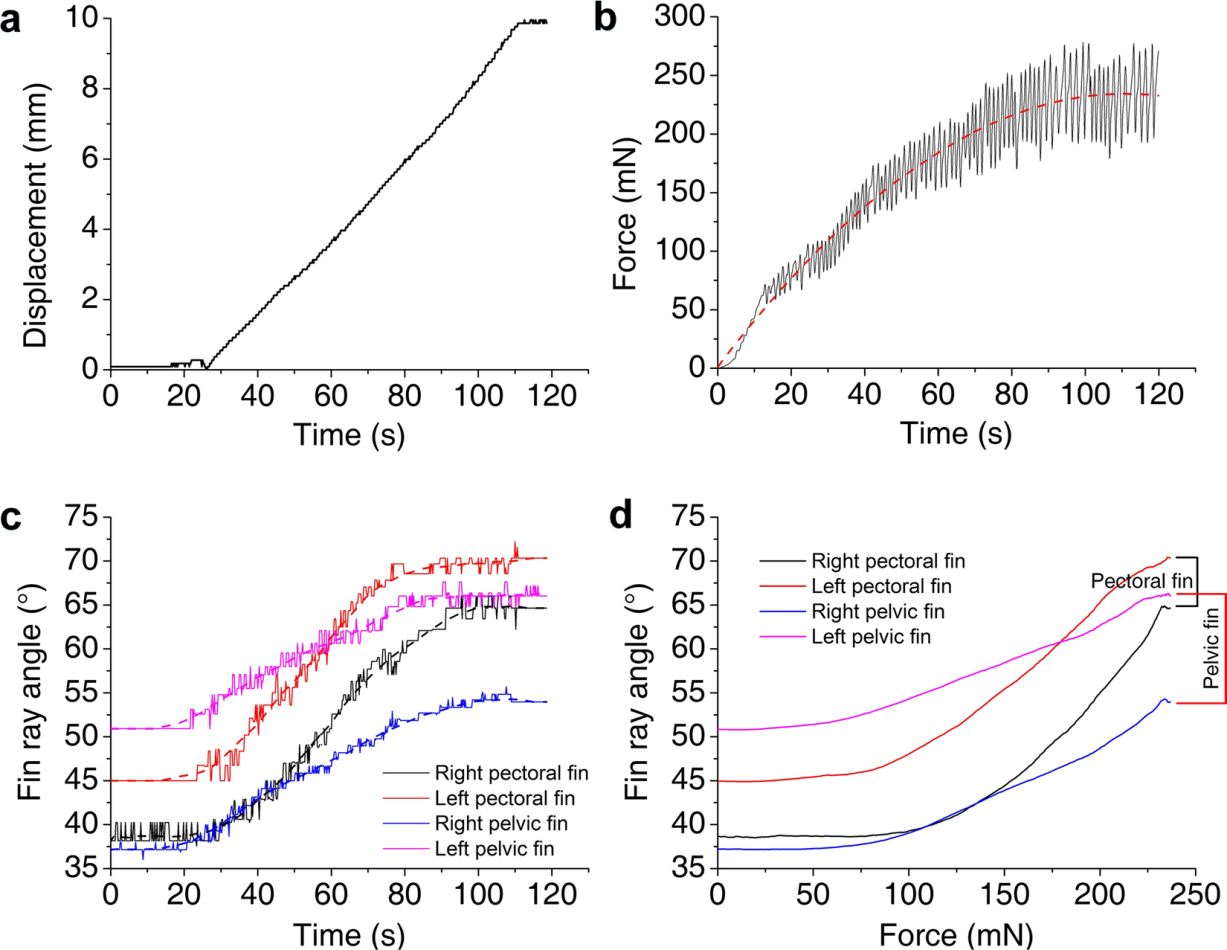
Friction force and fin ray angles underwater. (**a**) Displacement over time. (**b**) Force over time. The dashed line represents the smoothed fit line (using the Savitzky-Golay method). (**c**) Fin ray angle over time. The four dashed lines represent the smoothed fit lines (using the Savitzky-Golay method). (**d**) Fitting force as a function of fin ray angle.

Measurements were made on a smooth, transparent Plexiglas substrate with force gauge and high speed camera respectively recording force values and videos. Displacement was measured over time (Fig. 3a). The fish moved after 26 seconds, and reached maximum displacement (9.86 mm) after 111 seconds. Force was measured over time (Fig. 3b). After an initial increase in friction force (preceding a slip movement), the fish moved in a stick-slip fashion, as evidenced by rapid fluctuations in friction force. Friction force increased with time. Fin ray angle was measured over time (Fig. 3c). The angles of all fin rays, including those of the two pectoral fins and of the two pelvic fins, increased during movement, with the maximum angles observed at the end of the movement. Fitting force analysis indicated that the four fin ray angles increased after the fitting force reached ~65 mN (Fig. 3d).

### Fin and girdle muscle when crawling

A cycle was defined as the amount of time between the transition of the right pectoral fin (RF, right forefin) state, which from the ventral view could be seen to change abduction to adduction and back to abduction, or vice versa. Several cycles of crawling were observed. Crawling was indicated by slight undulations of the body axis, and the rapid alternate adduction and abduction of the pectoral and pelvic fins. During crawling, the pectoral and pelvic suckers were attached to the vertical surface. The rock-climbing fish rested pairs of fin on the substrate. The trunk of the fish curved alternately right and left. Although this motion was similar to that of swimming snakes and eels, the tail of the fish hardly moved. Instead, the paired fins were pulled in opposing directions by the trunk. For example, during crawling, the rock-climbing fish abducted the left pectoral fin and right pelvic fin, while simultaneously adducting the right pectoral fin and left pelvic fin. The fins are associated with the paired girdle muscles (the protractor and retractor).The contraction of the protractor pulls the pelvic fin forward and the pectoral fin back on the same side.

**Figure 4:**
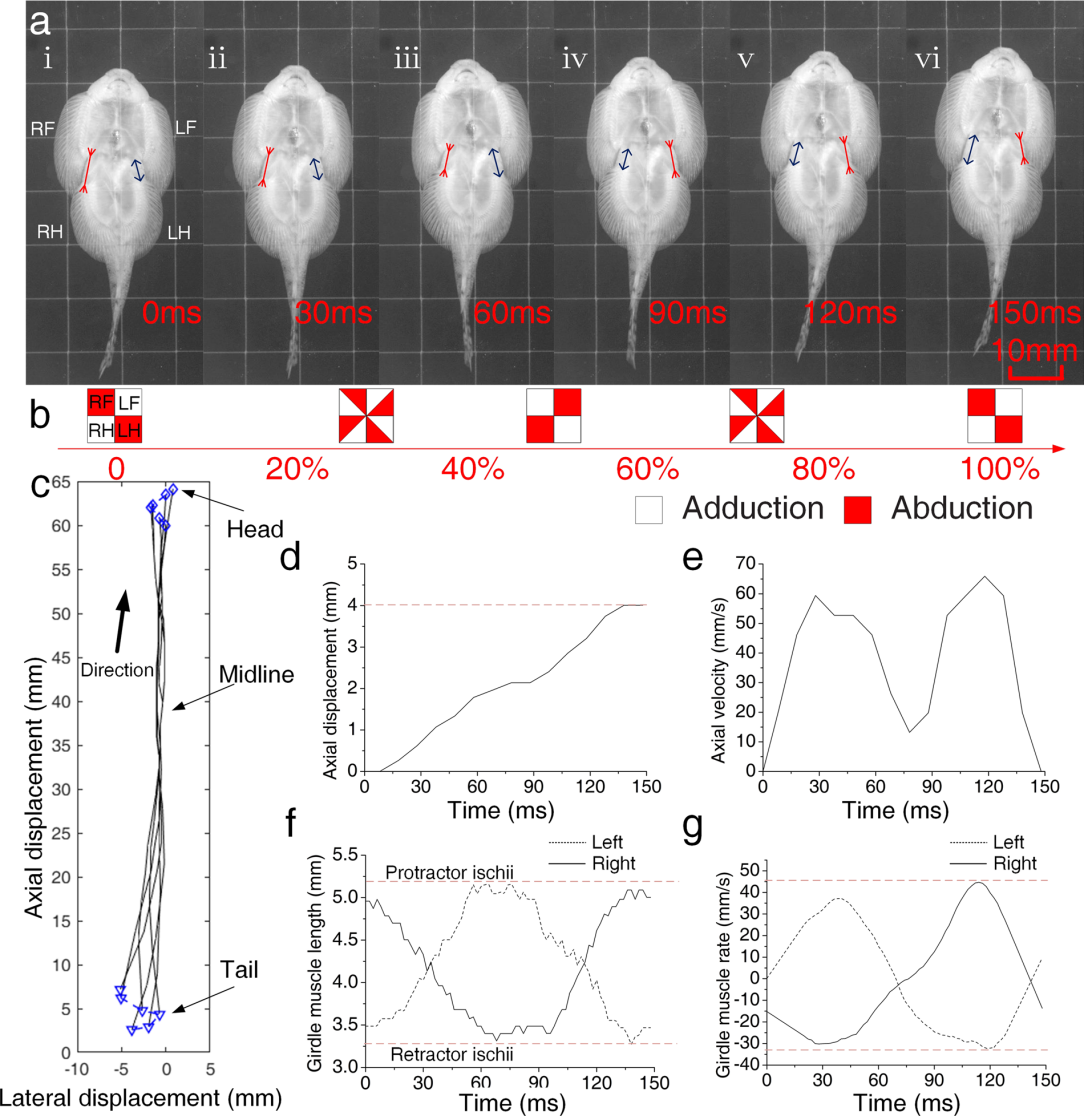
Kinematics during vertical forward crawling. (**a**) Sequential images of a forward crawling cycle. (**b**) Schematic diagram of fin states. Unfilled squares represent adduction, red squares indicate abduction, and half-red squares indicate a transitional state. (**c**) Sequential tracings of the midline from the head (blue diamond) to the tail (blue inverted triangle) through one complete crawling cycle. (**d**) Axial displacement. (**e**) Axial velocity. (**f**) Girdle muscle length. (**g**) Girdle muscle contraction rate. Right pectoral fin (RF, right forefin), left pectoral fin (LF, left forefin), right pelvic (RH, right hindfin), left pelvic (LH, hindfin), right girdle muscle (RG) and left girdle muscle (LG).

During forward crawling, the left pectoral fin transitioned from adduction to abduction, and then back to adduction (Fig. 4a; Supplementary Movie 1), the three other fins exhibited similar or inverse transformations (Fig. 4b). Based on fin transformations, we divided the crawling cycle into two distinct stages. Over the complete crawling cycle, the fish did not crawl in a straight line, but rather moved in a sinuous fashion, similar to the movements of a swing car (Fig. 4c). The maximum lateral displacement occurred at the end of the first stage. The movements of the main part of body axis were negligible, but those in the head and tail were somewhat more violent. During the first stage, the fish paused briefly, then crawled forward (up), first accelerating, then decelerating (Fig. 4d,e) (Supplementary Fig. 8). This process was repeated during the second stage. Similar to the fins, the left girdle muscle transitioned from a contracted state to a relaxed state, and then returned to a constricted state; the right girdle muscle exhibited an inverse contraction pattern (Fig. 4f,g). In addition, the changes in girdle muscle length also accelerated and decelerated twice. Girdle muscle contraction rate and axial velocity peaked simultaneously. Our observations indicated that the fish was able to sustain a maximum of 16 upward crawling cycles in one bout, and that climbing bout that lasted for 2.1 s. The fish crawled more than 130 mm in a rushing current (Supplementary Movie 2).

**Figure 5:**
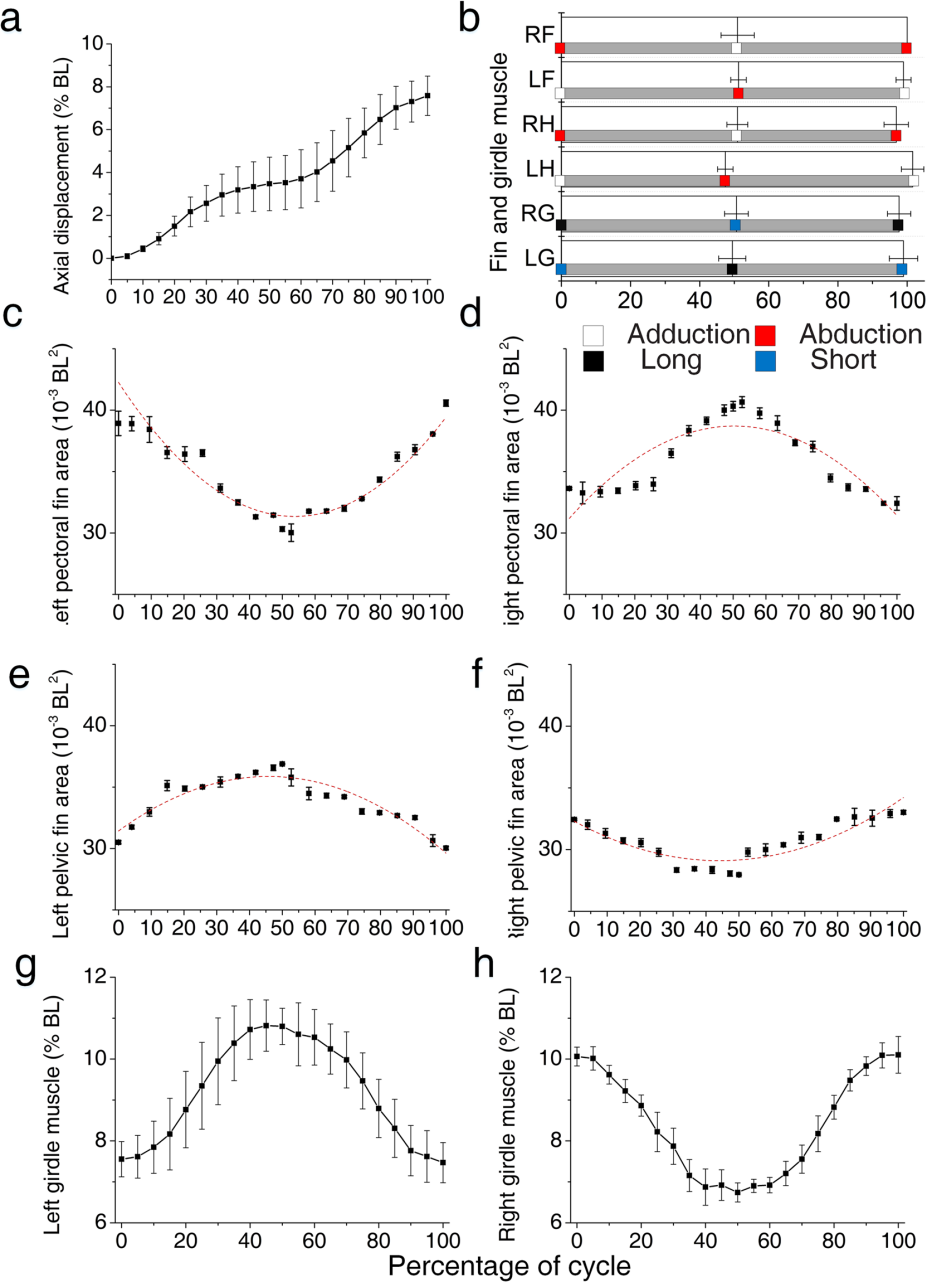
Mean profiles for kinematic variables. (**a**) Mean ± SD (n = 5 cycles) axial displacement. (**b**) Mean ± SD (n = 5 cycles) percentage of different states during the fin and girdle muscle transitions. The grey bar represents a transition state. (**c**–**f**) Mean ± SD (n = 5 cycles) areas of the four fins. Each quadratic polynomial fitting line is indicated as a dashed line. (**g**,**h**) Mean ± SD (n = 5 cycles) girdle muscle length. Right pectoral fin (RF, right forefin), left pectoral fin (LF, left forefin), right pelvic (RH, right hindfin), left pelvic (LH, hindfin), right girdle muscle (RG) and left girdle muscle (LG).

Each crawling cycle was normalized to the same duration, and values for the kinematic variables were interpolated at 20 equally spaced increments during the stroke cycle. We plotted the means (±SD) of each variable at 5% time increments throughout the crawling cycle. Because variations in the fin ray angle amplitudes of live fish are less obvious than those of dead fish, fin area was used to determine fin state. The crawling cycle began with decreases in the fin areas of the right pectoral and left pelvic fins, and increases in the fin areas of the other two fins (Fig. 5). These changes in area reflected changes in the abduction or adduction state of each fin. Concurrently, the right girdle transitioned from a relaxed state to a contracted state, and then returned to a relaxed state; the left girdle muscle exhibited an inverse contraction pattern. The areas of the right pectoral and the left pelvic fins were strongly positively correlated with the length of right girdle muscle (correlation coefficients are 0.95 and 0.86). Both left pectoral fin area and right pelvic fin area were obviously positively correlated with the length of the left girdle muscle (correlation coefficients of 0.95 and 0.85, respectively). The correlation coefficient between the right and left girdle muscles was about −1. Fin area was clearly associated with girdle muscle length, either positively or negatively. During crawling, the fish had two comprehensive fin and muscle states, as well as the transition state (Fig. 5b).

**Figure 6:**
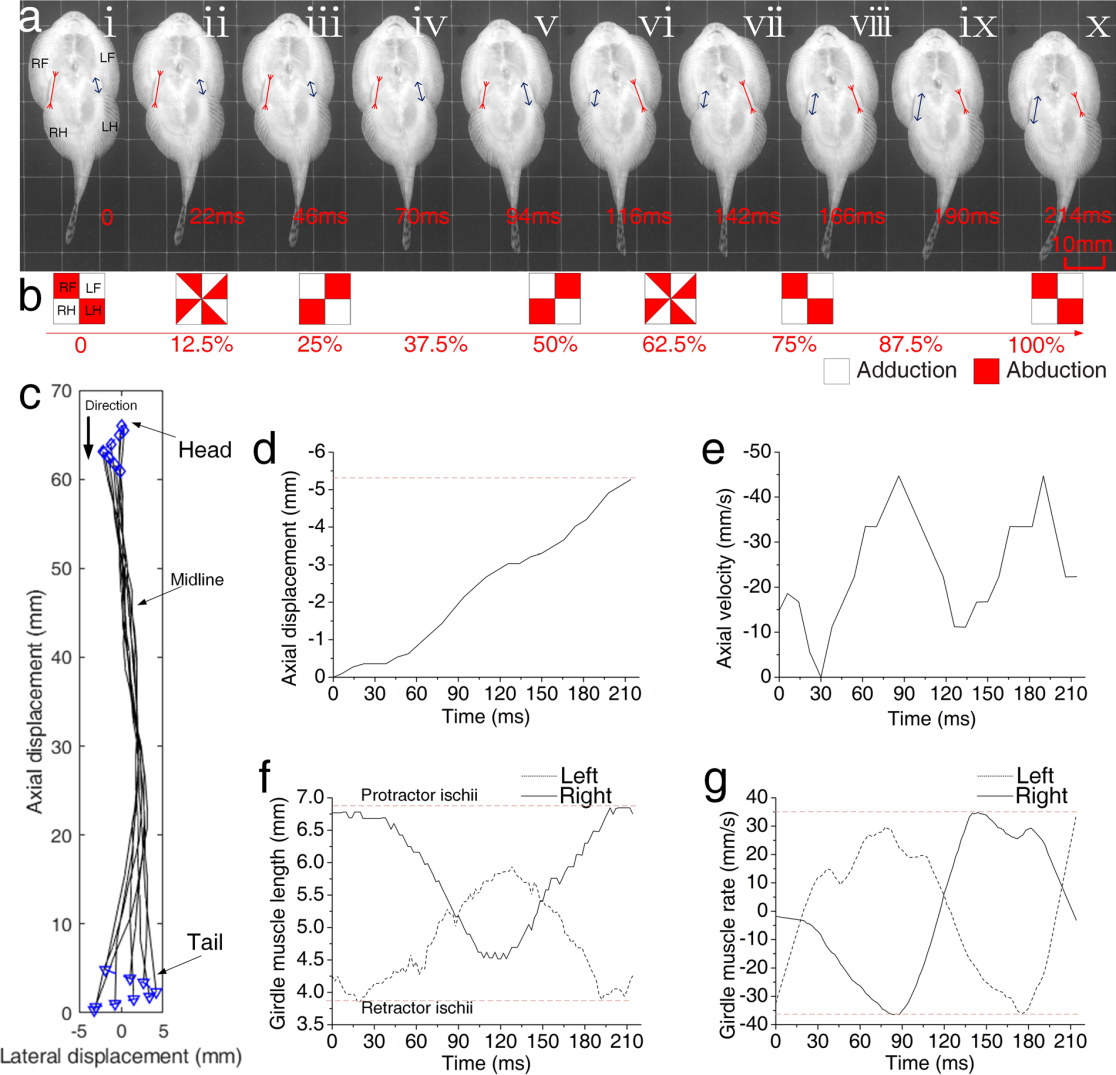
Kinematics during vertical backward crawling. (**a**) Sequential images of a backward crawling cycle. (**b**) Schematic diagram of fin states. Unfilled squares represent adduction, red squares indicate abduction, and half-red squares indicate a transitional state. (**c**) Sequential tracings of the midline from the head (blue diamond) to the tail (blue inverted triangle) through one complete crawling cycle. (**d**) Axial displacement. (**e**) Axial velocity. (**f**) Girdle muscle length. (**g**) Girdle muscle contraction rate.

As the rays of the fins touching the ground also moved back and forth, the fish were able to crawl backwards. During the 0–25% of the crawling cycle (first quarter), the left pectoral fin transitioned from adduction to abduction, and the three other fins exhibited similar or inverse transformations (Fig. 6a,b,d; Supplementary Movie 3) (Supplementary Fig. 8). However, the fish moved less than 0.5 mm. During the 25–50% of the crawling cycle (second quarter), the four fin states were similar to those observed during forward crawling, and the fish moved more than 2.5 mm. The first 50% including first and second quarters of the crawling cycle was considered the first stage, and the second 50% including third and forth quarters was considered the second stage. During the 50–75% of the crawling cycle, the four fins exhibited transitions. The third quarter was contrary to the first quarter of the cycle, particularly for the fin and girdle muscles. The second constant long displacement was observed during the forth quarter of the crawling cycle. The parameters were similar to those of the crawling forward cycle (Fig. 6c,e–g).

### Axial undulation kinematics during crawling

**Figure 7:**
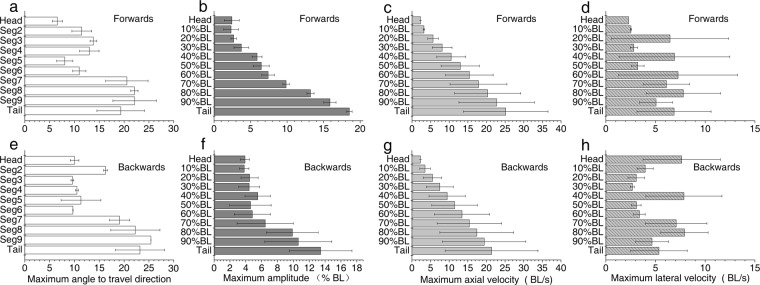
Axial kinematics during vertical forward and backward crawling. (**a**,**e**) Mean ± SD (n = 5 cycles) maximum angle of body segments with respect travel direction during forward and backward crawling. (**b**,**f**) Mean ± SD (n = 5 cycles) maximum amplitude of 11 equally spaced points along the midline during forward and backward crawling. (**c**,**g**) Mean ± SD (n = 5 cycles) maximum axial velocity of 11 equally spaced points along the midline during forward and backward crawling. (**d**,**h**) Mean ± SD (n = 5 cycles) maximum lateral velocity of 11 equally spaced points along the midline during forward and backward crawling.

The axial component of crawling was characterized by undulations of the whole body, resulting in maximum angles of all body segments relative to the direction of travel: a mean maximum of 6–22° per segment during forward crawling (n = 5 cycles), and a mean maximum of 9–26° per segment during backward crawling (n = 5 cycles) (Fig. 7).

When crawling forward, the smallest maximum segment angles occurred in the head, whereas the largest maximum segment angles occurred in the caudal peduncle (segments 8–9) (Fig. 7a). In addition, maximum amplitudes and velocities were high at all points along the midline of the body. Maximum amplitude and axial velocity increased along the midline, and largest amplitudes and axial velocities were observed in the tail. The largest amplitude was more than seven times the smallest amplitude (at the head) (Fig. 7b). Similar to the amplitude patterns, the largest axial velocity was about eleven times the smallest axial velocity (at the head) (Fig. 7c). The maximum lateral velocity varied up and down along the midline, with the largest lateral velocity (7.8 BL/s) at point 9 (located at 80% BL from the head) (Fig. 7d). The minimum axial and lateral velocities were both zero.

When crawling backward, the smallest maximum segment angles occurred in the pectoral sucking disc, whereas the largest maximum segment angles occurred in the tail segments (Fig. 7e). In addition, maximum amplitudes and velocities were high at all points along the midline of the body. Maximum amplitude was almost constant throughout the anterior of the fish, increasing at the posterior end of the midline; the largest amplitude and axial velocity were in the tail end. The largest amplitude was more than three times the smallest amplitude (near the head) (Fig. 7f). The largest axial velocity was more than nine times the smallest axial velocity (at the head) (Fig. 7g). The maximum lateral velocity varied up and down along the midline, with the largest lateral velocity (5.3 BL/s) observed in the head (Fig. 7h). The minimum axial and lateral velocities were both zero.

### Adhesive locomotion model

**Figure 8:**
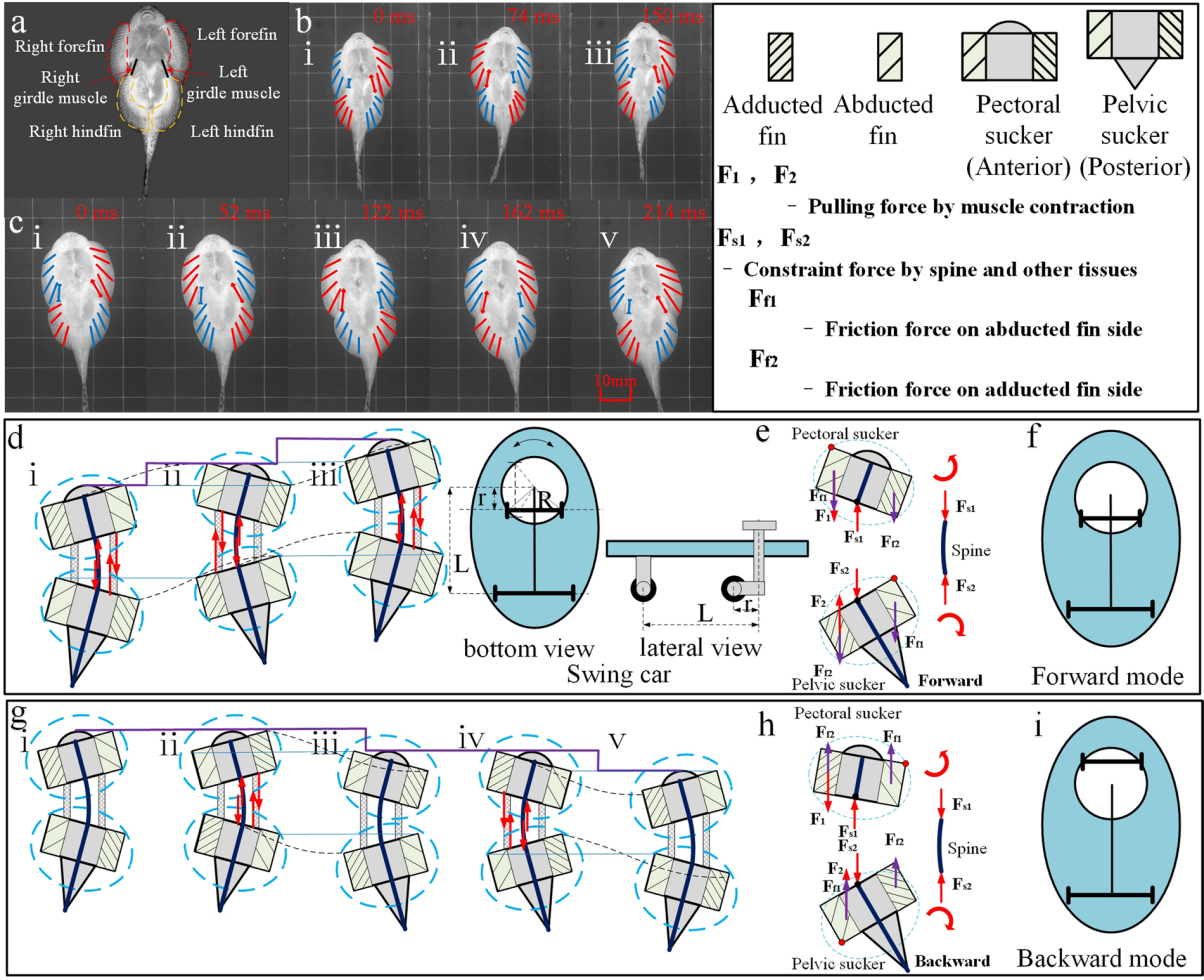
The bidirectional crawling and motion model. (**a**) The fins and girdle muscles. (**b**,**c**) Forward and backward crawling, red lines mean abducted fin, blue lines mean adducted fin, red line with arrows means protractor ischia, and blue line with arrows means retractor ischia.(**d**,**g**) Forward and backward crawling model. (**e**,**h**) Force analysis during forward and backward crawling. (**f**,**i**) Forward and backward mode of swing car. Purple lines trace the movement of the anterior-most point on the head; the black dashed lines trace the movements of the rotating fins; the solid blue lines trace the movements of the resting fins; the thick blue thick lines show the spine, which constrained the relative motions of the pectoral and pelvic suckers. The spine model was a bending bar of almost constant length. The figure is drawn by Jinrong Wang.

During locomotion, two suckers were distinguished; an anterior sucker, bearing the pectoral fins and the head, and a posterior sucker, bearing the pelvic fins. Based on our results, we designed a model of *B. kweichowensis* locomotion (Fig. 8). The motion model consisted of two anisotropic suckers (the anterior and posterior suckers) and two linear single-acting actuators (the girdle muscles). For the anterior anisotropic sucker, the sucker friction was influenced by the state of the bilateral pectoral fins. For the posterior anisotropic sucker, the sucker lateral friction was influenced by the state of the bilateral pelvic fins (Fig. 8a). Previous studies have suggested that friction is greater along the side of the body with abducted fins, than along the side of the body with adducted fins.

During the first stage, in the anterior section of the fish, the left side of the fish (with the abducted pectoral fin) generated the pulling force because of the contraction of the left relaxed girdle muscle (Fig. 8b). Due to the asymmetrical forces and unequal levels of friction along the sides of the fish, the posterior sucker rotated negatively around the right abducted pelvic fin. Due to morphological constraints (i.e., the spine and other tissues), the anterior sucker rotated positively around the left abducted pectoral fin. This propelled the fish forward in a crawling motion. During the rotation of the anterior and posterior suckers, abduction shifted to adduction, and vice versa (Fig. 8d(i,ii)). During the second stage, in the posterior section of the fish, the right side of the fish (with the adducted pelvic fin) generated the pulling force because of the contraction of the right relaxed girdle muscle. The posterior sucker rotated positively around the left abducted pelvic fin. Similarly, due to morphological constraints, the anterior sucker rotated negatively around the right abducted pectoral fin (Fig. 8d(ii,iii)). These changes cause the fish to crawl forward, returning to the original state. These two stages comprise one entire forward crawling cycle.

There were some differences between backward crawling and forward crawling. During the first quarter of backward crawling, abduction and adduction exchange was observed in all four fins, but there were almost no changes in muscle length or displacement (Fig. 8c). During the second quarter, in the anterior part of the fish, the left side of the fish (with the adducted pectoral fin) generated the pulling force because of the contraction of the left relaxed girdle muscle (Fig. 8g(ii)). Due to the asymmetrical forces and the unequal levels of friction on the sides of the fish, the anterior sucker rotated positively around the right abducted pectoral fin. Similarly, due to morphological constraints, the posterior sucker rotated negatively around the left abducted pelvic fin. This propelled the fish backward in a crawling motion. Unlike the forward crawling, abduction and adduction did not shift sides during the rotation of anterior and posterior suckers in backward crawling. However, muscle lengths changed and displacement was large. During the third quarter, only changes in fin state were observed; almost no displacement or changes in girdle muscle length were observed (Fig. 8g(iii,iv)). This was similar to the transition in first quarter. During the fourth quarter, in the anterior part of the fish, the left side of the fish (with the abducted pectoral fin) generated the pulling force because of the contraction of the right girdle muscle (Fig. 8g(iv)). Due to the asymmetrical forces and the unequal levels of friction on the sides of the fish, the anterior sucker rotated negatively around left abducted pectoral fin. Similarly, due to morphological constraints, the posterior sucker rotated positively around the right abducted pelvic fin. These changes caused the fish to crawl backward, transitioning to the last state(Fig. 8g(v))., which just like the initial stage (Fig. 8g(i)). These four repeating quarters comprised one entire backward crawling cycle.

The pelvic sucker was subjected to a pulling force (F_2_) due to muscle contraction and morphological constraints (F_s2_) (Fig. 8e,h). Due to the asymmetric pulling forces, the sucker had a tendency to rotate positively. Because the sucker was divided into two halves, the abducted side was harder to move than the adducted side, due to anisotropic friction. Thus, during the first stage, the adducted side moved first, and then the pelvic sucker rotated around the abducted side. After pelvic sucker rotation, the pulling force (F_1_) of the spine and morphological factors caused the pectoral sucker to rotate. Similarly, the adducted fin side moved earlier, and then the pectoral sucker rotated around the abducted side. Because all four fins transitioned from adduction to abduction (or vice versa) before muscle contraction, the backward crawling movement was the inverse movement of the forward crawling movement. *B. kweichowensis* and a swing car all can wriggle forward and backward just by a simple switch. *B. kweichowensis* switches its fin states from adduction to abduction (or vice versa), and swing car rotates its steering wheel for 180 degree to make it wriggle from forward to backward (more detail about swing car in Supplementary Fig. 11).

## Discussion

The fish that inhabit torrential streams may easily be swept away by the rapid and variable currents^2^. To survive under such conditions, the entire body of *B. kweichowensis* functions as an adhesive apparatus like *Beaufortia leveretti*^1^. Using an attachment disc sealed with a round belt of microbubbles and a passive mechanism supplying anisotropic shear force, a stationary *B. kweichowensis* on an underwater substrate resists a normal pulling force of up to 1000 times body weight, as well as a shear force of up to 100 times body weight^20^. Although the high resistance to shear force allows the fish to remain attached to the substrate, this strong adhesion may prevent efficient locomotion.

To explore the locomotion mechanisms used by *B. kweichowensis*, we investigated the skeletal mechanics of freshly euthanized fishes. As the anatomy of Balitorinae fish has previously been characterized in detail^1,25^, we focused on the bony plates and the fin ray locations only. The rock-climbing fish possessed two unconnected bony plates with paired fins; these were associated with the two individual suckers, without a rigid connection (Fig. 1). The rock-climbing fish had a similar number of pectoral fin rays to *Pseudogastromyzon myersi* (another species in the Balitorinae), which has 23 branched fin rays^1^. However, the number of pelvic fin rays was different in *B. kweichowensis* as compared to three other species of Balitorinae (*B. leveretti, Sewellia lineolate*, and *P. myersi*), all of which have 19 branched fin rays^1^.

To determine the influence of adhesion on friction, four different adhesion forces were tested using additional weight. The static friction force and the sliding friction force increased almost proportionally with adhesion; the friction coefficients were nearly constant. Because fin abduction affects shear force^20^, we hypothesized that fin ray angle influenced adhesion, and that adhesion in turn affected friction. Here, a stick-slip style of locomotion was evidenced by the observed rapid fluctuations in friction force (Fig. 3b). This was similar to the friction coefficients observed for natural and artificial remora spinules on rough glass substrates^11^. However, the microstructures of remora spinules suggest that these structures function using a ratcheting friction model, in which spinules interlock and slide over local asperities^11^. In contrast, the rock-climbing fish system is macroscopic, and, to date, no such property has been reported to be associated with the soft unculi. A second, ratchet-type locomotion mechanism has been described, in which local asperities are handled either during sliding (using dynamic friction) or at the beginning of the sliding motion (using static friction)^26,27^. The rock-climbing fish achieved similar results using a macroscopic approach. While the fish was stationary, maximum adhesion and friction force were determined by the fin ray state. Once the pulling force was maximized, the fin ray slipped slightly due to sliding friction, increasing adhesion and friction force because of the increase in fin ray angle. This continuous increase in adhesion with fin ray slippage constituted a unique, macroscopic, ratcheting friction model. Above a certain threshold, friction force increased with the four fin ray angles (Fig. 3d). Thus, friction was determined by adhesion, which was influenced in turn by fin ray angle (or by fin area, representing the degree of abduction). It should be noted that the experiments were done with euthanized fish, and fish may were operated far beyond their suffering stress in nature. So there may be something different from the fact in value, but the relationship between friction and adhesion is correct.

*B. kweichowensis* utilized the two anisotropic suckers with two paired fins and two girdle muscles during crawling. This undulatory motion was somewhat similar to the movement of climbing catfishes (*Astroblepus* sp. “Peru”), which crawl in a non-undulatory fashion; this movement is facilitated by an extremely mobile pelvic girdle^7^. Catfishes are known to climb the vertical rock faces of waterfalls using their mouths and pelvic fins in an alternating pattern^28^. However, sucker detachments have not been observed during rock-climbing fish crawling. In contrast to the straight path of the crawling catfish^7^, the path of the crawling rock-climbing fish, which moved using anisotropic suckers, was curved. Similar to catfishes, however, the crawling movement was driven by two girdle muscles: the retractor ischium and the protractor ischium^29,30^. The contraction of the protractor ischium on one side pulled the pelvic fin ahead or the pectoral fin back. Unlike other climbing fish with attachment discs, such as gobies and catfishes, *B. kweichowensis* locomotion was a combination of inching up and undulation, while maintaining constant adhesion. The maximum axial velocity of *B. kweichowensis* exceeded 65 mm/s (1.3 BL/s) during slow forward crawling, and 45 mm/s (0.9 BL/s) during slow backward crawling (Fig. 4b). The maximum axial velocity exceeded 103 mm/s (2.3 BL/s) during both forward and backward fast crawling. *Sicyopterus stimpsoni*, a goby with similar attachment discs, had a velocity of 6 mm/s (0.21 BL/s)^3^. The rock-climbing fish sustained 1.5 times more continuous climbing cycles as compared to inching-up climbing species; the cycle frequency of the rock-climbing fish (7.70 Hz) was also 1.75 times greater^3^. The crawling velocities observed for the rock-climbing fish (1.3–2.3 BL/s) were consistent with those at which red muscle fibers are typically recruited (<0.5–2 BL/s; while muscle fibers are recruited at 2–5 BL/s)^3^. This suggested that crawling in this fish probably depended on the activation of the red muscle fibers^31–33^. Thus, the potential for exhaustion might be greatly reduced in rock-climbing fish, because red muscle depends upon the aerobic metabolism, which is suited to endurance behaviors^3^. Meanwhile, *B. kweichowensis* accelerates along longitudinal axis that seems to be the coordinate of small friction compared to lateral axis due to the anisotropic property, hence the fish consumes less energy. These might explain the long distances traversed by *B. kweichowensis* during single crawling bouts. *B. kweichowensis* also used a launching motion to cope with sudden threats. Such high velocities (i.e., an initial velocity of 800 mm/s about 16 BL/s) allowed the fish to escape the threat, but were unsustainable (Supplementary Fig. 9). The forward crawling motion was typical (Fig. 5). During forward crawling, the fins and girdle muscles transitioned twice per cycle. The right pectoral fin and left pelvic fin transitioned synchronously with the right girdle muscle, while the left pectoral fin and right pelvic fin transitioned synchronously with left girdle muscle. During backward crawling, the sequence of fin and muscle transitions was altered: the fins transitioned before the muscles, reversing displacement. For lack of the actual force distributions experimental data, the model only refer to the visual locomotion video frame, but the model can explain the bidirectional locomotion well. More force data will reveal the locomotion mechanism more precisely.

During both forward and backward crawling, there were two forward or backward sucker rotations per cycle. During forward crawling, the fin states changed during sucker rotation (Fig. 5). In contrast, during backward crawling, the fin states changed before sucker rotation and remain unchanged during sucker rotation. There were three global states during the forward crawling cycle, but five global states during the backward crawling cycle. The backward crawling cycle differed from the forward cycle in that a fin state transition (between abduction and adduction) was required to reverse the direction of motion in two stages of the backward crawling cycle. During these two direction-reversal stages, almost no displacement was observed (Fig. 6d).

We compared the axial undulation kinematics of *B. kweichowensis* with those of gobies and eels (both of which use a powerburst style of locomotion)^3,13^. In gobies and eels, the body location with the lowest maximum amplitude corresponds to the location with the lowest minimum resultant velocity, but resultant velocities are high along the entire length of the body^3^. However, the minimum axial and lateral velocities in rock-climbing fish are zero. In rock-climbing fish, maximum amplitude also increased along the body midline. However, the maximum angle, axial velocity, and lateral velocity differed from those of gobies and eels. Although the rock-climbing fish crawled in an undulatory fashion, this movement was not based on an axial undulation mechanism. We also compared the locomotion mechanism of *B. kweichowensis* with the tetrapod-like mechanism used by mudskippers^15^, lungfish^16^ and cavefish^18^. The tetrapod-like mechanism in these fish was thought evolved during the invasion of land, and used on land rather than underwater. The gait duty factor was the proportion of the step cycle in which a fin was in contact with the substrate. The duty factor for African lungfish (*Protopterus annectens*) was highly variable, ranging from 0.06 to 0.82, with a mean of 0.46 ± 0.20^16^. The duty factor of cavefish (*Cryptotora thamicola*) was 65% for each fin with no significant difference among fins^18^. However, the fins of *B. kweichowensis* keep contact with the substrate (Fig. 5b), fins only change from abduction to adduction, or vice versa. Because the fish seals the suckers by the four fins, if one fin detaches from the substrate, the adhesion fails, and the fish will be swept away by the rush. Although solid-substrate based locomotion, alternating gait by pelvic propulsors, and digit-bearing limbs are considered to be critical adaptations for the emergence of tetrapods^16^. Our finding shows that fish are capable of evolving to climb on solid-substrate in a diagonal-couplets lateral sequence gait underwater in the absence of digited limbs, more specifically is in a free bidirectional way. Our work supports that pelvic appendage driven locomotion on a hard surface was possible before the evolution of digited limbs^16^.

Based on our results, we developed a locomotion model for the adhesive system of *B. kweichowensis* (Fig. 8). Two anisotropic suckers, along with the paired girdle muscles, formed a unique system for crawling locomotion. This system allowed the rock-climbing fish to crawl forwards and backwards while maintaining adhesion (i.e., without detaching either sucker). In addition, the powerful tail of the rock-climbing fish facilitated an escape mechanism with a high initial velocity (Supplementary Figs 9 and 10). In other species, including tree frogs^34,35^, snakes^36,37^, remoras^11^, and sharks^38^, microstructures provide anisotropic friction against the substrate. When used in legged-robot locomotion, similar microstructures reduce energy requirements, but are difficult to fabricate and easily damaged^33^. Our model demonstrates a novel, macroscopic mechanism for the generation of anisotropy, and provides a framework for the development of new devices for real-world applications.

In conclusion, we identified a unique system of locomotion, based on adhesive undulation, in the rock-climbing fish. The locomotion system comprised two anisotropic suckers (associated with the pectoral and pelvic fins) and two girdle muscles. The fin states controlled the direction of motion, and the girdle muscles provided power during crawling. However, the tail generated the propulsive force for launching. This adhesive locomotion system was very effective on underwater vertical surfaces, and allowed the fish to move without detaching from the substrate. This mechanism may represent an energy-saving biomimetic solution to the problem of climbing while adhering to vertical or inclined surfaces. However, current methods is not functional directly, further investigations are required, including an analysis of the stress distributions under the two ventral suckers during locomotion and the electromyography of the pelvic and pectoral muscles.

## Methods

### Animals

The experiments herein were approved by the animal welfare committee of Zhejiang University (Hangzhou, China) and all the experimental trials were carried out in accordance with the relevant guidelines and regulations.

We obtained 12 Chinese rock-climbing fishes^19^ (60.3 ± 3.9 mm standard length) from a commercial suppler. Four of the fish were euthanized via submersion in 0.5 g/L MS-222 (Sigma Chemical, USA), and scanned using µCT, as described by Zou *et al*.^20^. We examined the relationship between friction force and adhesion force, and between friction force and fin angle. The eight remaining live fish were kept in a single aquarium (50 cm × 30 cm × 44 cm) with an oxygen pump, a filter, and a heater. The aquarium was maintained at 26 °C. The aquarium substrate was loose sand (~5 cm deep) as well as stones on which the fish could rest. Fish were typically observed adhering to the stones or the aquarium walls; fish shifted location only occasionally. During the acclimation period, fish were fed fish food three times a week.

### Friction force measurement

We measured friction force using the freshly euthanized fish. We used a digital force gauge (HBO HF-50) with a screw platform system to generate a constant pulling speed of 0.1 mm/s. The force data generated by the digital force gauge was visualized and collected in Microsoft Excel. We attached each freshly euthanized fish to the force gauge with suture thread. The thread was looped under the vertebral column near the caudal end of the suction disc and through the opercular gill openings^2^. The fish was gently pressed to remove any water from under the sucker disc before friction force measurement. The screw platform system pulled the fish along the tail.

To test the relationship between friction force and adhesion force, we used a smooth, transparent Plexiglas plate (10 cm × 10 cm) as the substrate. The plate was positioned such that the upper surface was at a depth of 10 cm. Three fixed pulleys (each 24 mm in diameter) were used to adjust the cord direction with respect to the weight tray and the force gauge (Supplementary Fig. 1). The total weight of the weight tray and the additional weights were considered the adhesion force, and the load value of the force gauge was considered the friction force.

To test the relationship between friction force and fin angle, a high-speed camera (Phantom V2512) and a LED light source were used to record fin ray angle changes. Each fish was pressed onto the wall of a transparent Plexiglas container, with the ventral side of the fish facing the camera. The fish remained submerged throughout the experiment (Supplementary Fig. 4). The videos were saved as Cine and JPEG files for subsequent analysis^3^.

### Locomotion kinematics

**Figure 9:**
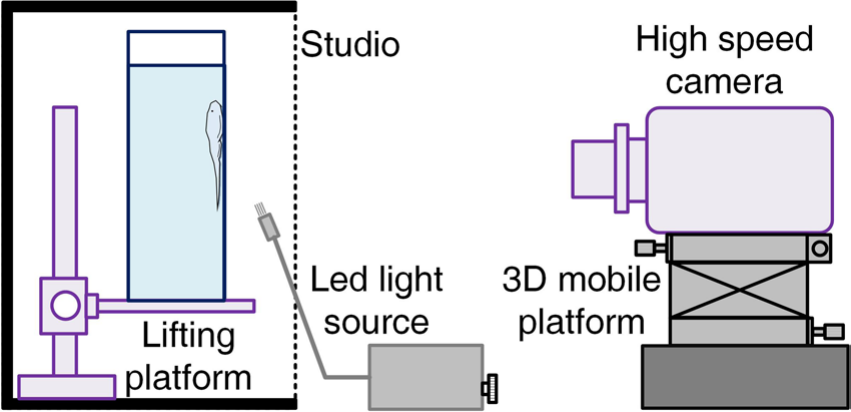
The experimental set up for the observation of locomotion kinematics. The figure is drawn by Jinrong Wang.

We constructed a simple photo studio in order to film the locomotion of the rock-climbing fishes. We used a transparent Plexiglas container, the surface of which was engraved with gridlines (1 mm deep) outside, a high-speed camera (Phantom V2512), and a LED light source (Fig. 9). The transparent surface allowed us to observe the kinematics of the pectoral fin, pelvic fin, and muscles on the ventral side of each fish. In the wild, water-worn rocks may be very smooth, and rock-climbing fish are frequently observed climbing smooth rock and concrete obstacles in natural stream habitats. Plexiglas substrates were therefore appropriate.

To evaluate locomotion kinematics, three types of video recordings of rock-climbing fish locomotion were collected in the ventral view using a high-speed camera (Phantom V2512; frame rates 500 and 1000 Hz). Videos were saved as Cine and JPEG files.

### Data analysis

To calculate the axial kinematics, 20 points were digitized random along the midline of the fish for every frame of a locomotor cycle, with the first point marking the anteriormost extent of the head and the last point marking the caudal peduncle. Identification of the midline was facilitated by the presence of a white line running anterior to posterior along the fish ventral surface except the girdle area. For each frame, 101 equally spaced points were interpolated from the 20 digitized points with the software Origin (OriginLab Origin 8.0). From these interpolated points, the coordinates were extracted of 11 evenly spaced points along the length of the body to divide the body into 10 segments, each of which was 10% of body length. To aid evaluations of axial mechanics during locomotion, custom software was developed in Matlab (Mathworks) to calculate the following parameters for every frame of each locomotor cycle analysed from the coordinates of the 11 equally spaced midline points and the orientations of the 10 equal body segments: (1) the angle of each body segment to the direction of travel; (2) the amplitude of each landmark point relative to a straight body midline; (3) the instantaneous resultant velocity of each landmark point (Supplementary Fig. 5). Measurements of segment angles and undulatory amplitudes along the body were performed to help identify which parts of the fish contribute to thrust generation. In order to reflect the motion of the whole fish, 10 points including first point marking the anteriormost extent of the head and the last point marking the tip of the tail were collected in ImageJ (National Institutes of Health) in some frame in a locomotor cycle^5^.

To quantify fin movement, the bases and tips of the left and right pectoral and pelvic fins were digitized for each frame of sequences of fin motion, along with 2 points along the midline of the head to serve as a reference for the instantaneous direction of travel. By µCT technology, there are 24 or 25 fin rays in pectoral fin and 20 fin rays in pelvic fin. The two terminals of middle fin rays was taken as a reference for the base and tip. For example, the two terminals of the 12th left pectoral fin ray was taken as a reference for left pectoral fin motion (Supplementary Fig. 5). Besides, two additional points in the anterior midline of the fish serve as a reference for anterior sucker instantaneous direction, and two additional points in the posterior midline serve as a reference for posterior sucker instantaneous direction.

From these coordinates, the angle of each fin relative to the direction of travel was calculated for each frame, allowing evaluation of the synchronicity of left and right fin movements. Due to the irregular shape of the fin, the fin areas were calculated in ImageJ for each frame(Supplementary Fig. 6). These data allowed us to evaluate when the fins moved and, therefore, when they might contribute to thrust generation during each locomotion cycle.

To quantify pelvic muscle movement, two pelvic muscles are prominently visible through the ventral skin, and extension or contraction of the pelvic muscles switched alternately. In order to explore the propulsion, 2 points were digitized for each frame of sequences of locomotor circle each pelvic muscle in ImageJ (National Institutes of Health) (Supplementary Fig. 7). From these coordinates, the length of two muscles was calculated for each frame, allowing evaluation of the transformation of left and right pelvic muscles.

The data used to support the findings of this study are available from the corresponding author upon request.

## Supplementary information

backward locomotion video

upstream locomotion video

forward locomotion video

Supplementary material
