# Supplementary material for: An adhesive locomotion model for the rock-climbing fish, Beaufortia kweichowensis
Source: Sci Rep. 2019 Nov 12;9:16571. doi: 10.1038/s41598-019-53027-2 (PMC6851163; doi:10.1038/s41598-019-53027-2)
Supplement: Supplementary file 4 — Supplementary material [file 41598_2019_53027_MOESM4_ESM.pdf]

# Supplementary material

# An adhesive locomotion model for the rock-climbing fish, *Beaufortia kweichowensis*

Jinrong Wang[1], Chen Ji[2], Wei Wang[1], Jun Zou[1*], Huayong Yang[1], Min Pan[3]

[1] State Key Laboratory of Fluid Power and Mechatronic Systems, Zhejiang University, Hangzhou 310027, China

[2] Institute of Marine Science and Technology, Shandong University, Qingdao 266237, China

[3]Center for Power Transmission and Motion Control, Department of Mechanical Engineering, University of Bath, BA2 7AY, UK

*Correspondence and requests for materials should be addressed to J. Z. (junzou@zju.edu.cn)

## Friction force at different adhesion force

We performed friction force measurement with the fresh euthanized fish. We used a digital force gauge (HBO HF-50) with a screw platform system at a constant pulling speed of 0.1 mm/s. When force measurement were applied in the water, three fixed pulley 24 mm in diameter was needed to adjust the cord direction. We attached freshly euthanized fish to force gauge with suture thread that looped under the vertebral column near the caudal end of the suction disc and through the opercular gill openings. The substrate is a 10 cm× 10 cm smooth transparent acrylic plate, and the substrate upper surface is 10 cm below the water.

At one adhesion force condition, three friction forces were tested.

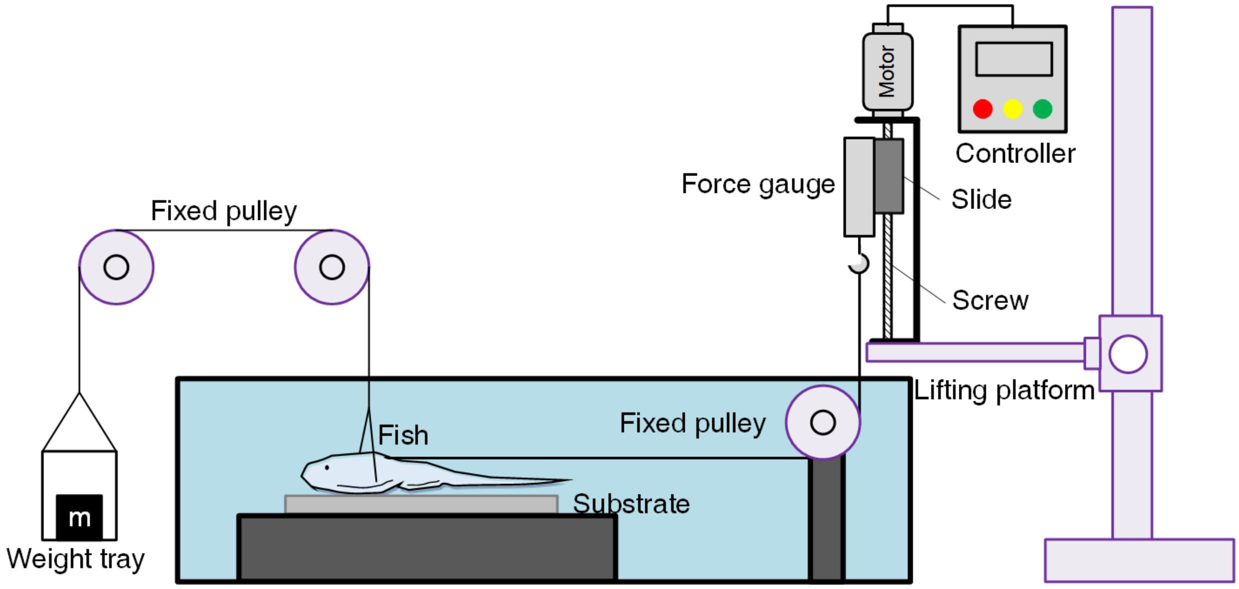


**Figure 1 | Experimental setup about shear force and normal force underwater.** The gravity of weight tray is about 0.06 N, and the gravity of aluminium block weight is 0.1 N each. The screw platform system ran at a constant pulling speed of 0.1 mm/s. The combination of weight tray and weight blocks refers to the adhesion force. The figure is drawn by Jinrong Wang.

**Table 1. Data of static friction force at different adhesion force**

| adhesive force (N) | static friction force (N) | | | | |
|---|---|---|---|---|---|
| | min | middle | max | mean | sd |
| 0.06 | 0.13 | 0.15 | 0.17 | 0.15 | 0.01633 |
| 0.16 | 0.25 | 0.28 | 0.3 | 0.276667 | 0.020548 |
| 0.26 | 0.42 | 0.47 | 0.48 | 0.456667 | 0.026247 |
| 0.36 | 0.7 | 0.76 | 0.8 | 0.753333 | 0.041096 |

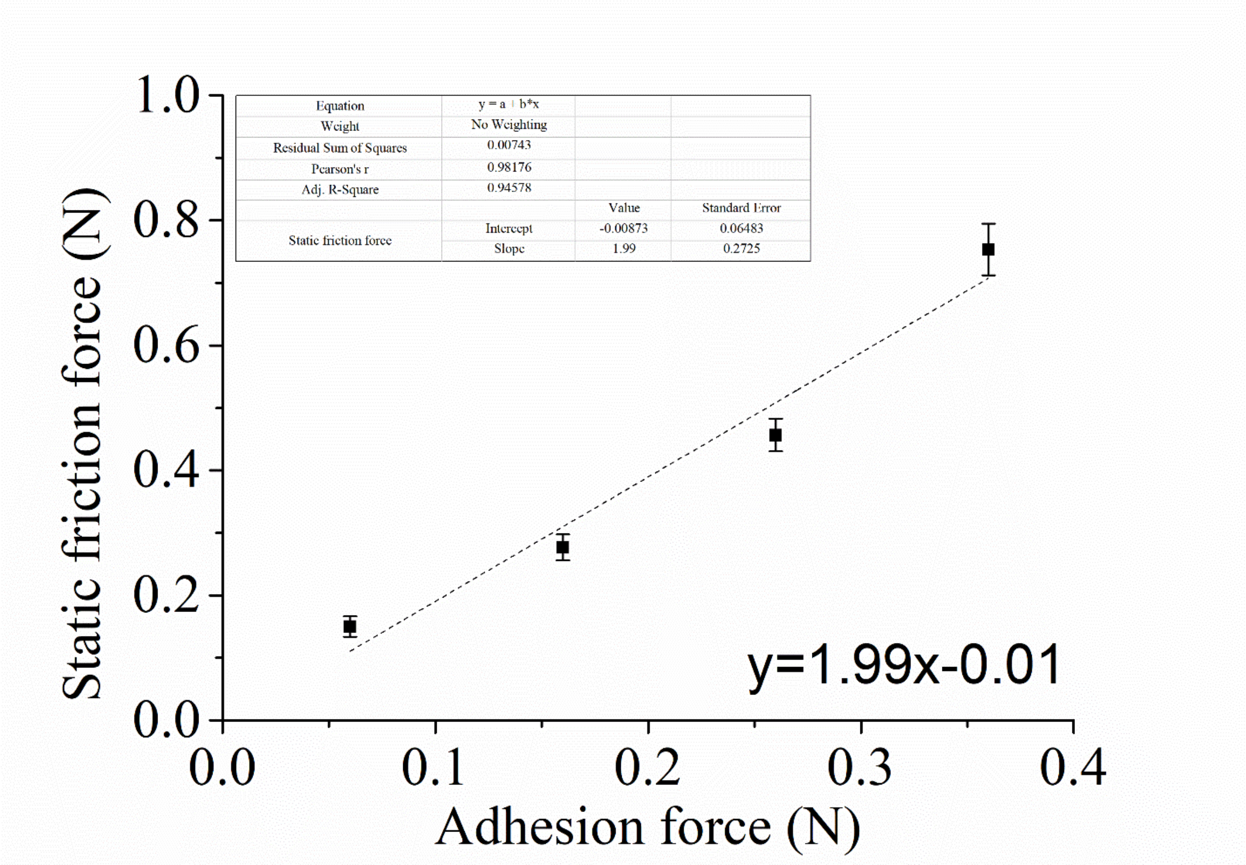


**Figure 2 | Static friction forces at different adhesion force on underwater substrate.** An expected line for relationship between static friction and adhesion force is indicated as a dotted line. The slope of the dotted line refers to the static friction coefficient.

**Table 2. Data of sliding friction force at different adhesion force**

| adhesive force(N) | slinding friction force(N) | | | | |
|---|---|---|---|---|---|
| | min | middle | max | mean | Sd |
| 0.06 | 0.07 | 0.08 | 0.1 | 0.083333 | 0.012472 |
| 0.16 | 0.18 | 0.19 | 0.2 | 0.19 | 0.008165 |
| 0.26 | 0.25 | 0.28 | 0.29 | 0.273333 | 0.016997 |
| 0.36 | 0.39 | 0.44 | 0.45 | 0.426667 | 0.026247 |

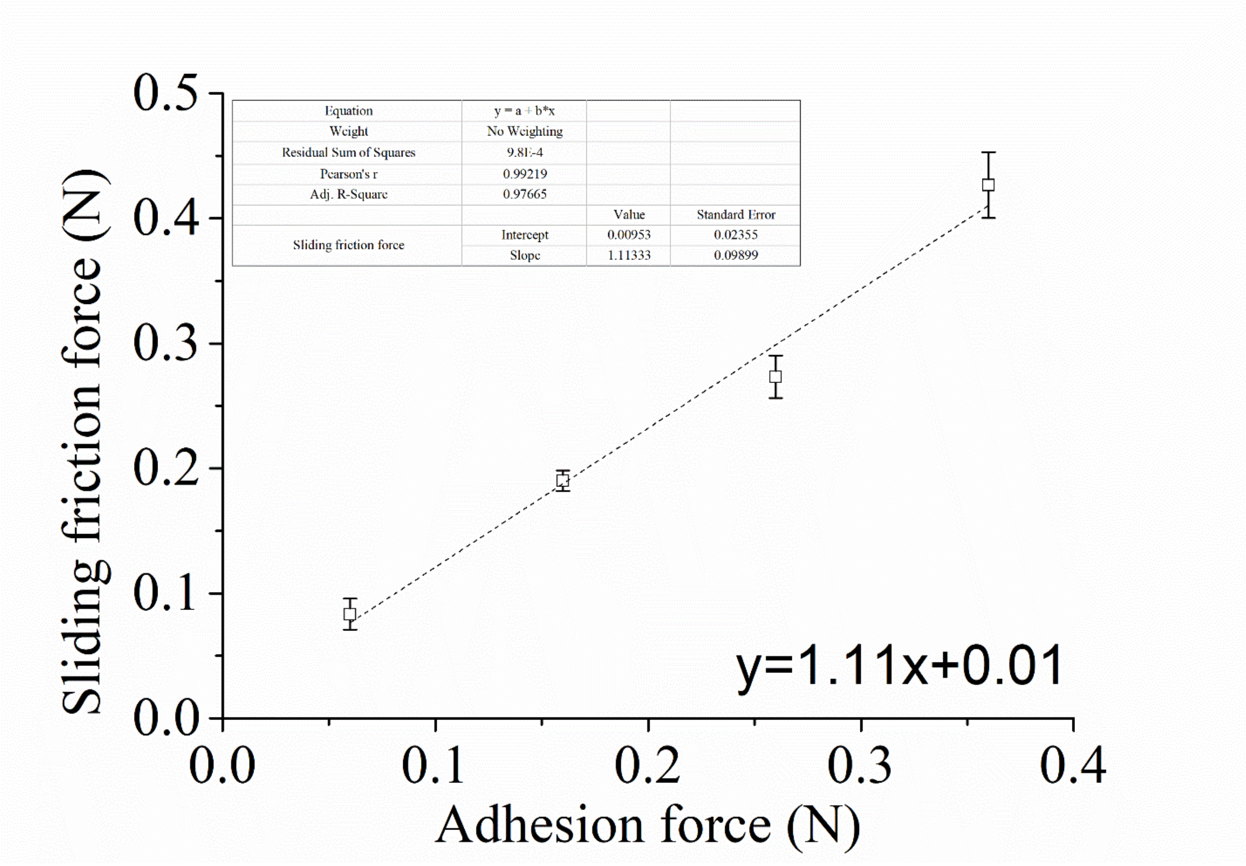


**Figure 3 | Sliding friction forces at different adhesion force on underwater substrate.** An expected line for relationship between sliding friction and adhesion force is indicated as a dotted line. The slope of the dotted line refers to the sliding friction coefficient.

## Friction force and fin ray angles during pulling backwards

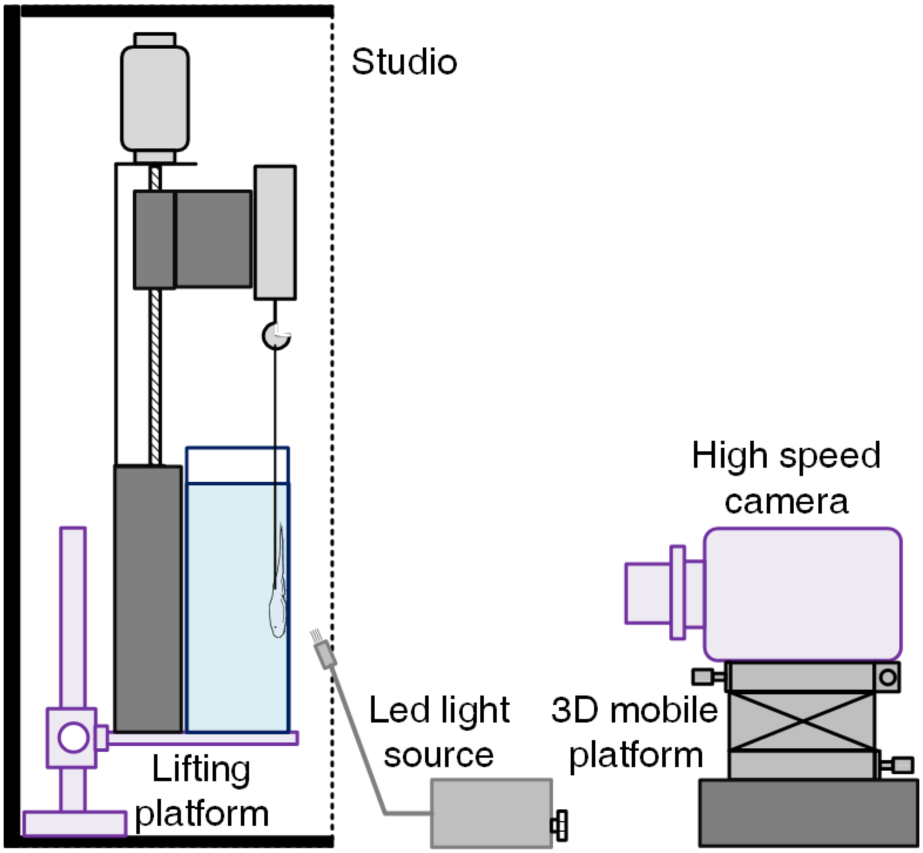


**Figure 4 | Experimental setup about friction force and fin ray angles underwater.** The figure is drawn by Jinrong Wang.

## Locomotion kinematics analysis

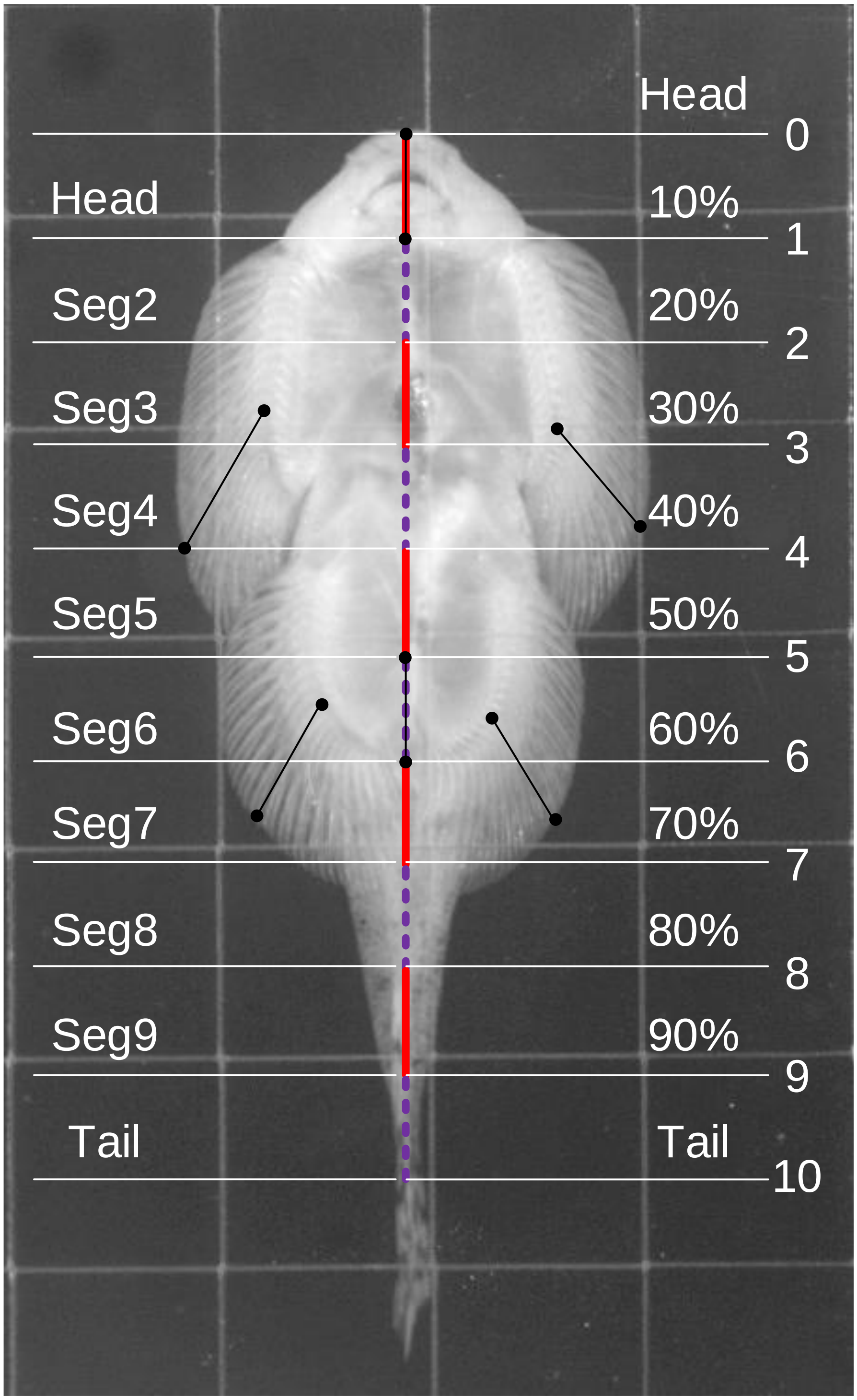


**Figure 5 | Digitized points of interest in the fish.** Points about 12th pectoral fin ray and 10th pelvic fin ray, and 10 equally spaced midline points.

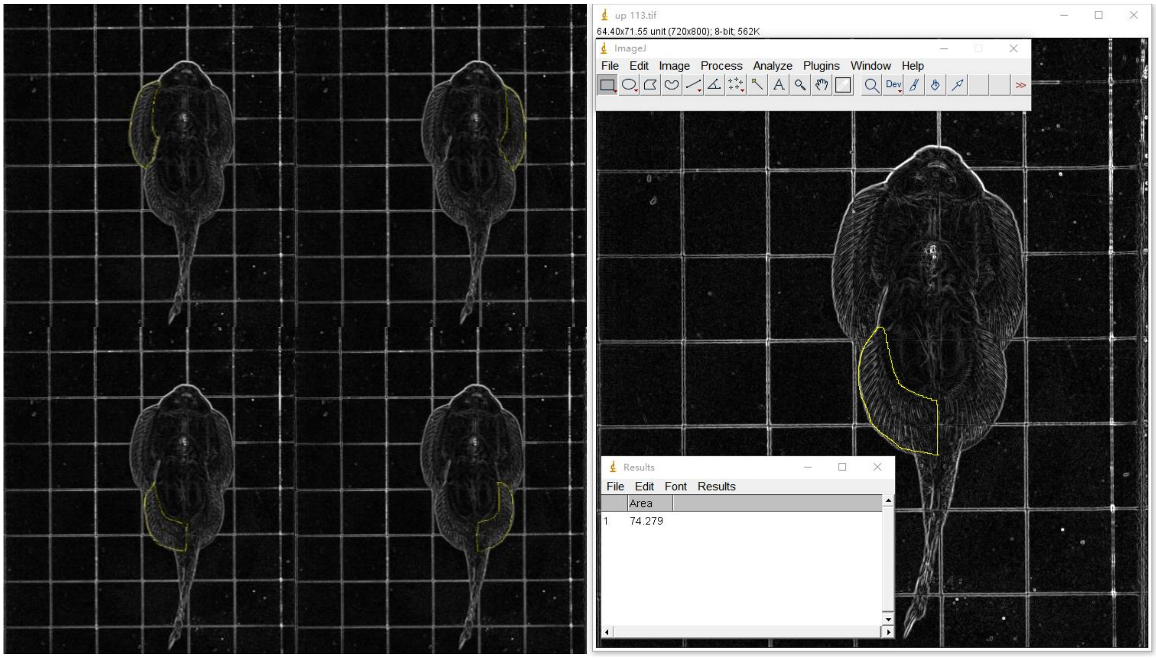


**Figure 6 | Approach to calculate the fin area.**

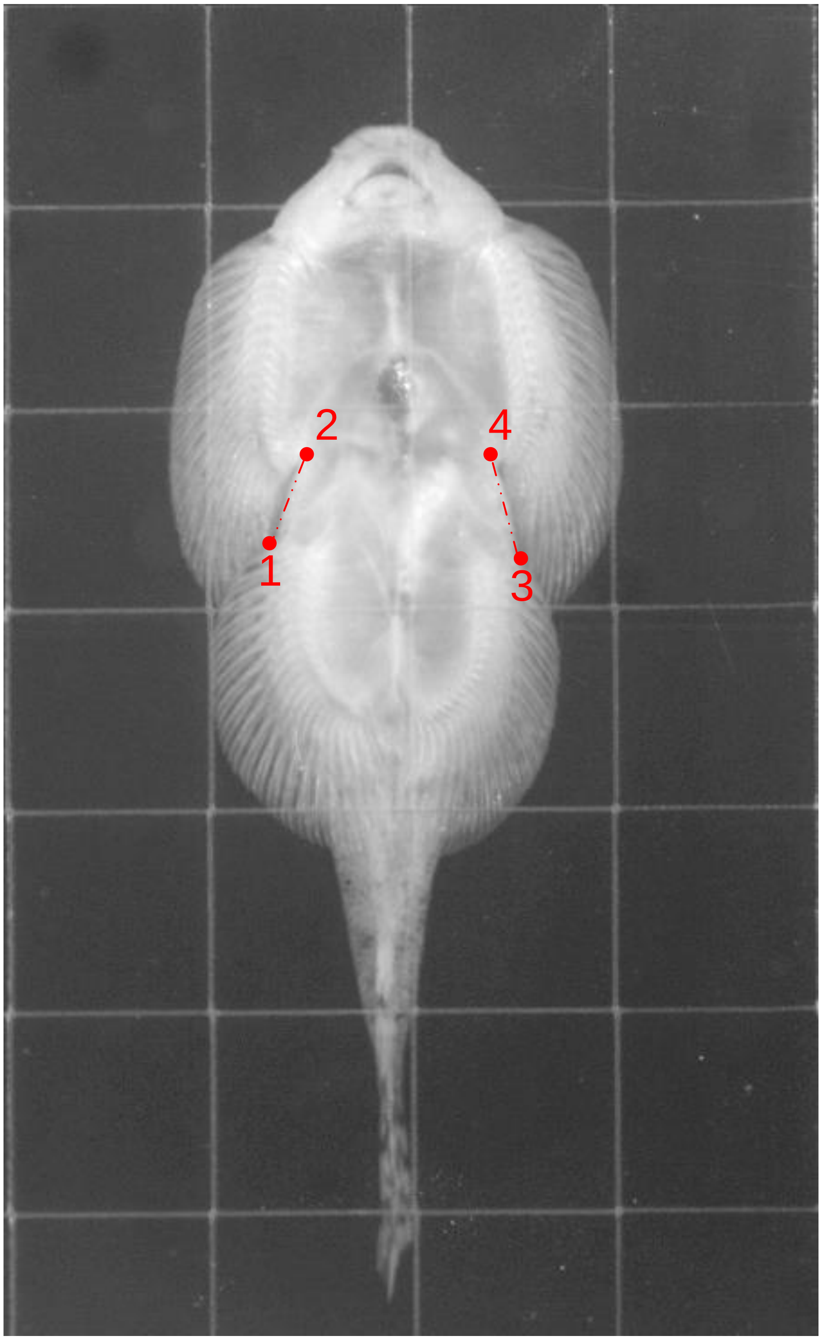


**Figure 7 | Digitized points of interest in the fish.** Points about girdle muscle.

## Path of forward and backward crawling

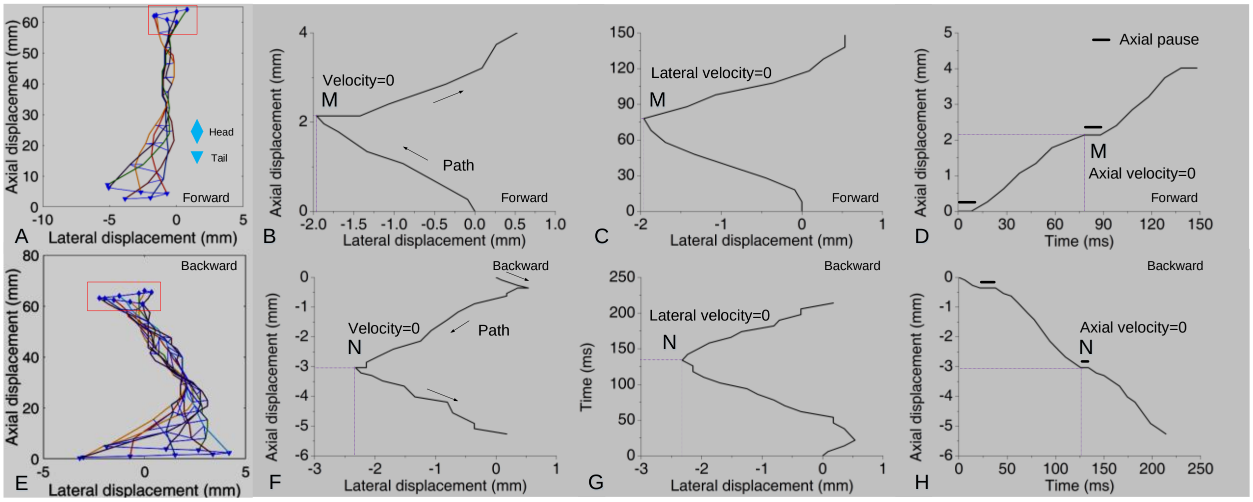


**Figure 8 |** Sequential tracings in bidirectional crawling. (A) and (E) Sequential tracings of the midline with 10 points along the midline. (B-D) and (F-G) Head path, lateral and axial displacement.

## A locomotion different from the adhesive undulatory mechanism (launching)

They quickly hold all fins convergent, and beat the tail once, make the body launch like a bullet when suffer external stimulus.

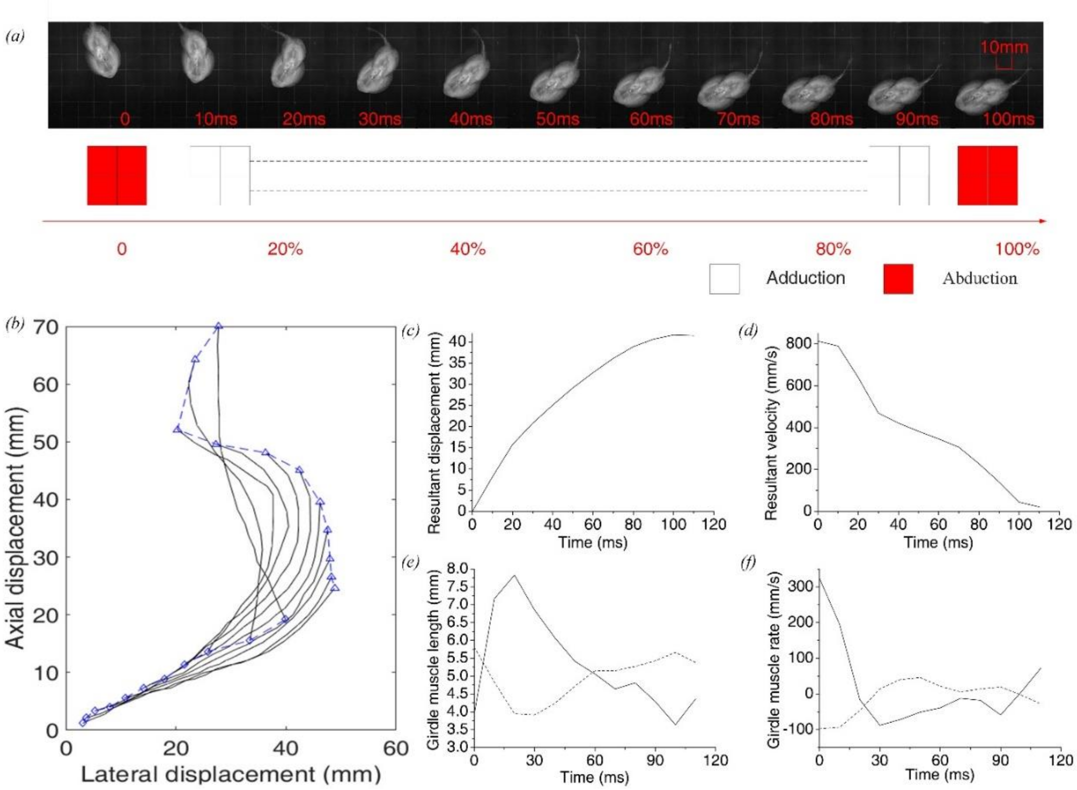


**Figure 9 | tail push motion.**

## Adhesive locomotion model (included the launching)

During locomotion, two suckers were distinguished; an anterior sucker, bearing the pectoral fins and the head, and a posterior sucker, bearing the pelvic fins. Based on our results, we designed a model of *B. kweichowensis* locomotion (Fig. 10). The motion model consisted of two anisotropic suckers (the anterior and posterior suckers), two linear single-acting actuators (the girdle muscles), and a propeller (the tail). For the anterior anisotropic sucker, the sucker friction was influenced by the state of the bilateral pectoral fins. For the posterior anisotropic sucker, the sucker lateral friction was influenced by the state of the bilateral pelvic fins. Previous studies have suggested that friction is greater along the side of the body with abducted fins, than along the side of the body with adducted fins.

During the first stage, in the anterior section of the fish, the right side of the fish (with the abducted pectoral fin) generated the pulling force because of the contraction of the right girdle muscle (Figs 10a–b). Due to the asymmetrical forces and unequal levels of friction along the sides of the fish, the posterior sucker rotated positively around the left abducted pelvic fin. Due to morphological constraints (i.e., the spine and other tissues), the anterior sucker rotated negatively around the right abducted pectoral fin. This propelled the fish forward in a crawling motion. During the rotation of the anterior and posterior suckers, abduction shifted to adduction, and vice versa. During the second stage, in the posterior section of the fish, the left side of the fish (with the adducted pelvic fin) generated the pulling force because of the contraction of the left girdle muscle. The posterior sucker rotated negatively around the right abducted pelvic fin. Similarly, due to morphological constraints, the anterior sucker rotated positively around the left abducted pectoral fin. These changes cause the fish to crawl forward, returning to the original state. These two stages comprise one entire forward crawling cycle.

There were some differences between backward crawling and forward crawling. During the first quarter of backward crawling, abduction and adduction exchange was observed in all four fins, but there were almost no changes in muscle length or displacement (Figs 10c–d). During the second quarter, in the anterior part of the fish, the right side of the fish (with the adducted pectoral fin) generated the pulling force because of the contraction of the right girdle muscle. Due to the asymmetrical forces and the unequal levels of friction on the sides of the fish, the anterior sucker rotated negatively around the left abducted pectoral fin. Similarly, due to morphological constraints, the posterior sucker rotated positively around the right abducted pelvic fin. This propelled the fish backward in a crawling motion. Unlike the forward crawling, abduction and adduction did not shift sides during the rotation of anterior and posterior suckers in backward crawling. However, muscle lengths changed and displacement was large. During the third quarter, only changes in fin state were observed; almost no displacement or changes in girdle muscle length were observed. This was similar to the transition in first quarter. During the fourth quarter, in the anterior part of the fish, the left side of the fish (with the abducted pectoral fin) generated the pulling force because of the contraction of the left girdle muscle. Due to the asymmetrical forces and the unequal levels of friction on the sides of the fish, the anterior sucker rotated positively around right abducted pectoral fin. Similarly, due to morphological constraints, the posterior sucker rotated negatively around the left abducted pelvic fin. These changes caused

the fish to crawl backward, transitioning to the last state， which just like the initial stage. These four repeating quarters comprised one entire backward crawling cycle.

Fins remained adducted to minimize friction, and the tail provided a powerful propulsive force by beating strongly (Figs 10e–f). It was likely that the girdle muscle on one side remained contracted, resulting in a curved route.

For example, the pelvic sucker was subjected to a pulling force ($F_2$) due to muscle contraction and morphological constraints ($F_{s2}$) (Fig. 10a, c, e). Due to the asymmetric pulling forces, the sucker had a tendency to rotate positively. Because the sucker was divided into two halves, the abducted side was harder to move than the adducted side, due to anisotropic friction. Thus, during the first stage, the adducted side moved first, and then the pelvic sucker rotated around the abducted side. After pelvic sucker rotation, the pulling force ($F_1$) of the spine and morphological factors caused the pectoral sucker to rotate. Similarly, the adducted fin side moved earlier, and then the pectoral sucker rotated around the abducted side. Because all four fins transitioned from adduction to abduction (or vice versa) before muscle contraction, the backward crawling movement was the inverse movement of the forward crawling movement. During launching, the adduction of all fins minimized friction, the contraction of the girdle muscle inclined movement direction to one side, and the tail provided power.

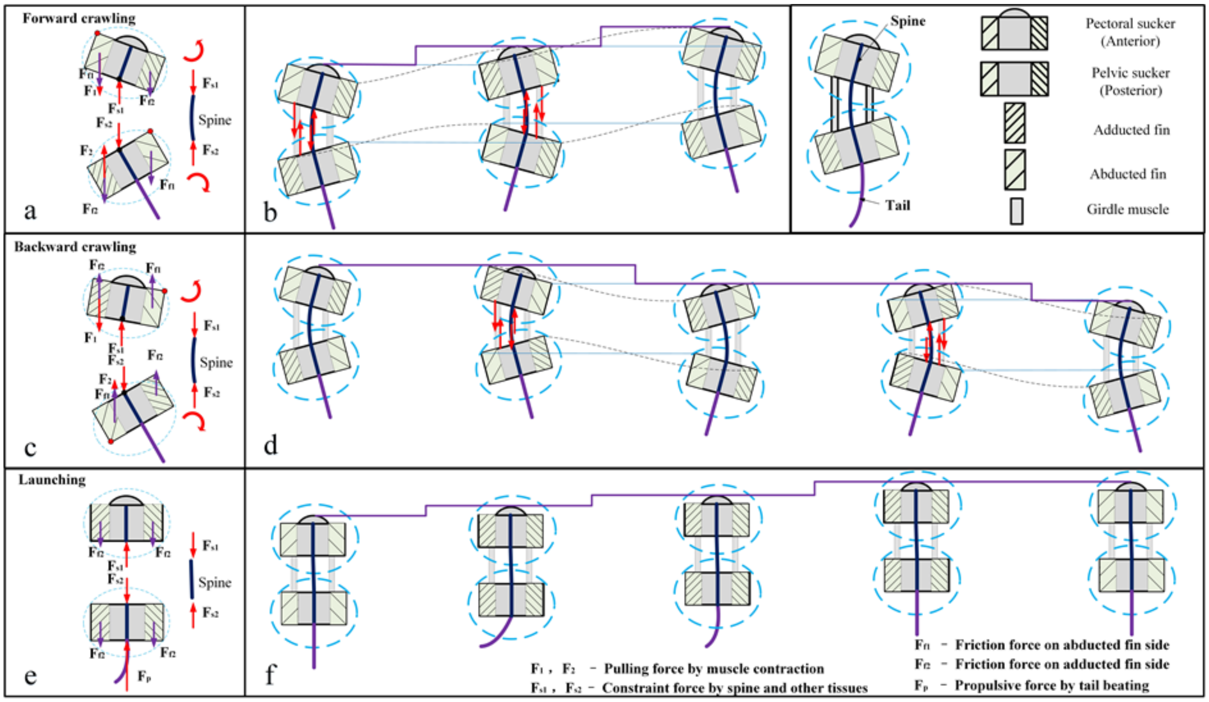


**Figure 10 | motion model.** The figure is drawn by Jinrong Wang.

## A swing car

We define the distance between line through two forewheel centers and line through two hindwheel centers as λ.

(a) Forward mode: λ = L – r (see the illustration at top-right). If driver rotates the steering wheel clockwise or counterclockwise, the swing car will wriggle forward.
(b) Backward mode: λ = L + r (see the illustration at bottom-right). If driver rotates the steering wheel clockwise or counterclockwise, the swing car will wriggle backward.

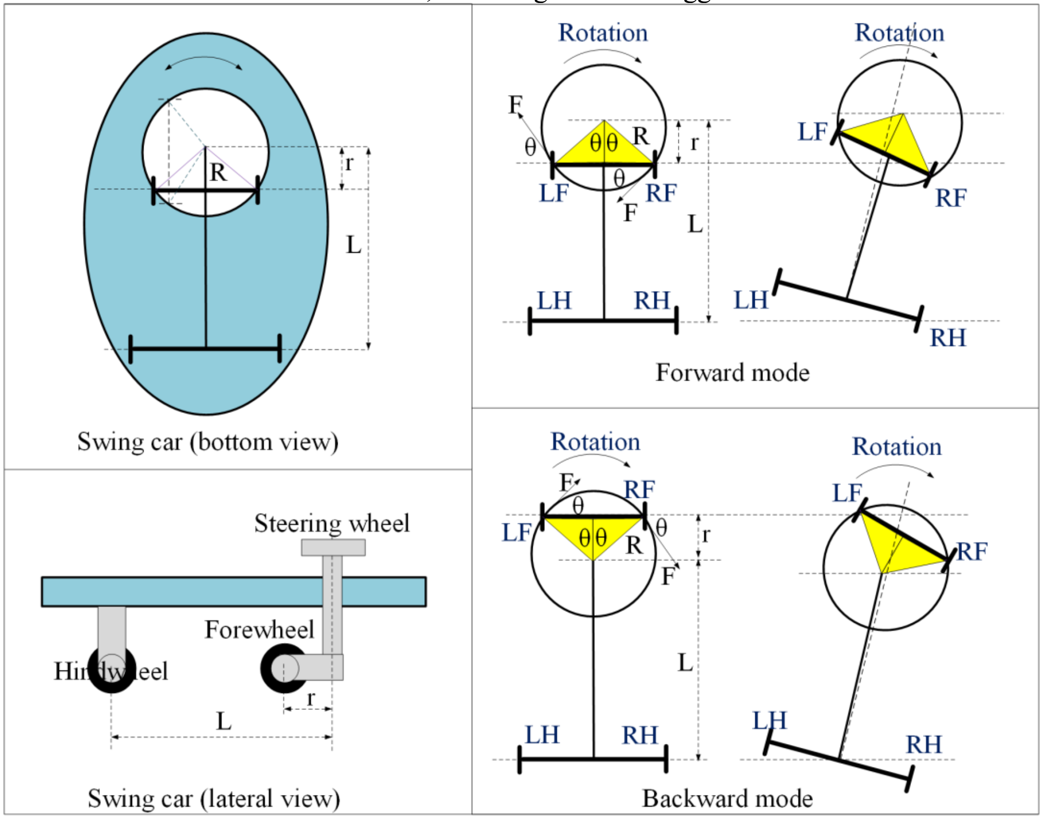


**Figure 11 | swing car.** The figure is drawn by Jinrong Wang.

**Table 3 | Mean data for kinematic variables**

| Axial displacement | Right pectoral fin area | Left pectoral fin area | Right pelvic fin area | Left pelvic fin area | Right girdle muscle length | Left girdle muscle length |
|---|---|---|---|---|---|---|
| 0 | 38.92398 | 33.62346 | 30.50109 | 32.42986 | 10.06279 | 7.55316 |
| 0.09824 | 38.89636 | 33.25841 | 31.74255 | 32.01066 | 10.01627 | 7.61342 |
| 0.4454 | 38.42672 | 33.35972 | 32.97771 | 31.31018 | 9.61755 | 7.84544 |
| 0.90462 | 36.54676 | 33.43943 | 35.12374 | 30.71766 | 9.22075 | 8.16695 |
| 1.49515 | 36.42125 | 33.85138 | 34.88324 | 30.55606 | 8.86477 | 8.76491 |
| 2.16517 | 36.5052 | 33.96896 | 35.01313 | 29.77837 | 8.22091 | 9.34589 |
| 2.56218 | 33.64753 | 36.48988 | 35.41756 | 28.34427 | 7.86967 | 9.94661 |

| | | | | | | |
|---|---|---|---|---|---|---|
| 2.95023 | 32.48455 | 38.33183 | 35.86874 | 28.4434 | 7.15369 | 10.3861 |
| 3.18524 | 31.30216 | 39.13508 | 36.19264 | 28.34687 | 6.87249 | 10.72225 |
| 3.33499 | 31.45023 | 40.00137 | 36.57616 | 28.05934 | 6.92083 | 10.81732 |
| 3.46735 | 30.31679 | 40.31747 | 36.88173 | 27.96254 | 6.74158 | 10.79839 |
| 3.52821 | 30.01723 | 40.65436 | 35.79054 | 29.79382 | 6.90021 | 10.60427 |
| 3.70662 | 31.75991 | 39.75048 | 34.47908 | 29.99754 | 6.92085 | 10.53099 |
| 4.02004 | 31.77167 | 38.93793 | 34.30667 | 30.38406 | 7.20499 | 10.24522 |
| 4.53724 | 31.99945 | 37.34892 | 34.21725 | 30.96185 | 7.55466 | 9.97954 |
| 5.16049 | 32.78343 | 37.03719 | 33.01441 | 31.01203 | 8.17472 | 9.46802 |
| 5.84504 | 34.34345 | 34.47881 | 32.91482 | 32.4609 | 8.82455 | 8.79377 |
| 6.46934 | 36.21466 | 33.71097 | 32.67569 | 32.64602 | 9.48038 | 8.30798 |
| 7.01745 | 36.77796 | 33.57533 | 32.51777 | 32.54361 | 9.82798 | 7.76107 |
| 7.30559 | 38.06043 | 32.41054 | 30.63741 | 32.91605 | 10.09059 | 7.61908 |
| 7.5812 | 40.57342 | 32.40443 | 30.02734 | 33.01709 | 10.10341 | 7.46947 |

**Table 4 | Correlation analysis**

| | Right pectoral fin area | Left pectoral fin area | Right pelvic fin area | Left pelvic fin area | Right girdle muscle length | Left girdle muscle length |
|---|---|---|---|---|---|---|
| Right pectoral fin area | 1 | | | | | |
| Left pectoral fin area | -0.948946027 | 1 | | | | |
| Right pelvic fin area | -0.799182213 | 0.751898401 | 1 | | | |
| Left pelvic fin area | 0.744248128 | -0.779537224 | -0.921024275 | 1 | | |
| Right girdle muscle length | 0.951956928 | -0.954432625 | -0.858018996 | 0.859307296 | 1 | |
| Left girdle muscle length | -0.953278543 | 0.95095505 | 0.854753135 | -0.865300449 | -0.996265788 | 1 |
